# Non-viral gene delivery to human mesenchymal stem cells: a practical guide towards cell engineering

**DOI:** 10.1186/s13036-023-00363-7

**Published:** 2023-07-25

**Authors:** Natalia Carballo-Pedrares, Federica Ponti, Junquera Lopez-Seijas, Diego Miranda-Balbuena, Nina Bono, Gabriele Candiani, Ana Rey-Rico

**Affiliations:** 1 Gene & Cell Therapy Research Group (G-CEL). Centro Interdisciplinar de Química y Biología - CICA, Universidade da Coruña, As Carballeiras, S/N. Campus de Elviña, 15071 A Coruña, Spain; 2 genT_LΛB, Department of Chemistry, Materials and Chemical Engineering “G. Natta”, Politecnico Di Milano, 20131 Milan, Italy; 3 Laboratory for Biomaterials and Bioengineering, Canada Research Chair I in Biomaterials and Bioengineering for the Innovation in Surgery, Department of Min-Met-Materials Engineering & Research Center of CHU de Quebec, Division of Regenerative Medicine, Laval University, Quebec City, QC Canada

**Keywords:** Mesenchymal stem cells, Non-viral gene delivery vectors, Tissue engineering, Regenerative medicine, Osteochondral repair

## Abstract

In recent decades, human mesenchymal stem cells (hMSCs) have gained momentum in the field of cell therapy for treating cartilage and bone injuries. Despite the tri-lineage multipotency, proliferative properties, and potent immunomodulatory effects of hMSCs, their clinical potential is hindered by donor variations, limiting their use in medical settings. To address this challenge, gene delivery technologies have emerged as a promising approach to modulate the phenotype and commitment of hMSCs towards specific cell lineages, thereby enhancing osteochondral repair strategies. This review provides a comprehensive overview of current non-viral gene delivery approaches used to engineer MSCs, highlighting key factors such as the choice of nucleic acid or delivery vector, transfection strategies, and experimental parameters. Additionally, it outlines various protocols and methods for qualitative and quantitative evaluation of their therapeutic potential as a delivery system in osteochondral regenerative applications. In summary, this technical review offers a practical guide for optimizing non-viral systems in osteochondral regenerative approaches.

**Graphical Abstract:**

hMSCs constitute a key target population for gene therapy techniques. Nevertheless, there is a long way to go for their translation into clinical treatments. In this review, we remind the most relevant transfection conditions to be optimized, such as the type of nucleic acid or delivery vector, the transfection strategy, and the experimental parameters to accurately evaluate a delivery system. This survey provides a practical guide to optimizing non-viral systems for osteochondral regenerative approaches.

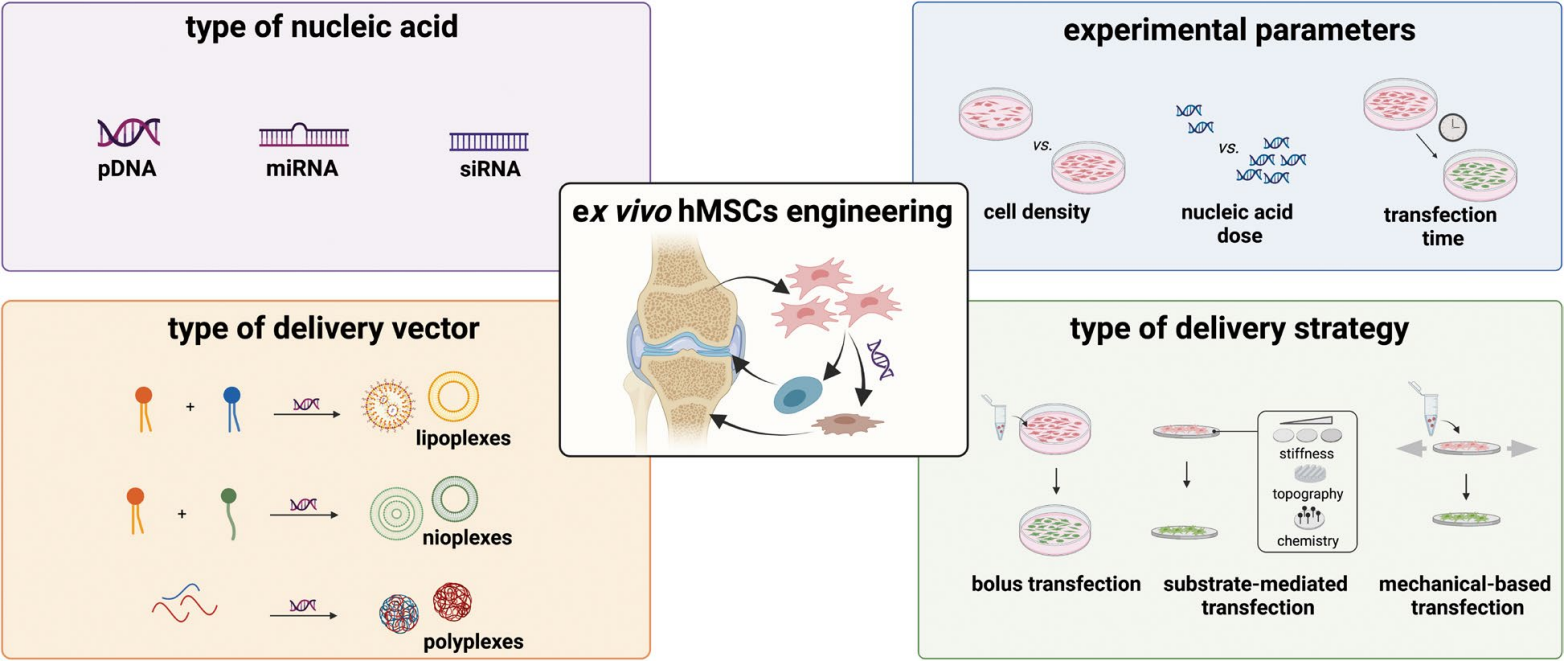

**Supplementary Information:**

The online version contains supplementary material available at 10.1186/s13036-023-00363-7.

## Highlights

- hMSCs-based therapies represent a promising strategy for osteochondral regeneration and repair;

- Non-viral gene delivery vectors are a promising and safe tool to engineer hMSCs* ex vivo*;

- There is a lack of standardized procedures to engineer hMSCs* ex vivo*;

- Optimization of key transfection parameters is needed to effectively engineer hMSCs.

**Supplementary Information:**

The online version contains supplementary material available at 10.1186/s13036-023-00363-7.

## Introduction

In the last decades, human mesenchymal stem cells (hMSCs) have gained momentum in several cell therapy applications for the treatment of cartilage and bone lesions [1–4]. hMSCs are multipotent stem cells that can be isolated and expanded from many tissues, such as bone marrow (hBMMSCs) [5], umbilical cord (hUCMSCs) [6], and adipose tissue (hAMSCs) [7], possess high capacity for self-renewal, display a potent immunomodulatory effect in vivo, and under appropriate culture conditions display a multi-lineage multipotency differentiation in vitro (i.e., they can differentiate into osteoblasts, adipocytes, chondrocytes, myocytes, and neurons). All these characteristics make hMSCs a very appealing tool for tissue engineering, regenerative medicine approaches [5], and the treatment of many diseases and disorders including cardiovascular diseases [8], autoimmunity [9], and cancer [7].

However, the aging of hMSCs, which relies on donor age, is a critical factor affecting cell therapy outcomes, primarily when used in cartilage tissue repair strategies [10]. In this context, the possibility to modulate the phenotype and commitment of hMSCs toward a selective cell lineage using gene delivery technologies has paved the way for the development of ever more effective osteochondral repair strategies [11]. To date, conventional approaches relying on the use of recombinant growth factors (GFs) have significant drawbacks such as the short half-life of proteins, and rapid body clearance. This implies repeated administrations to achieve and lengthen the therapeutic effect of the treatment [12]. In this scenario, gene delivery strategies based on the delivery of osteo or chondrogenic genes, such as those encoding for the insulin-like growth factor-1 (IGF-1) [13–15], transforming growth factor beta (TGF-β) [16–19], bone morphogenic proteins (BMPs) [20–23], runt-related transcription factors (RUNXs) [24–26], as well as genes encoding for the SOX family transcription factors [27–29] among others, did improve the regenerative potential of hMSCs (for comprehensive reviews, please refer to [30–32]). Although various approaches have proven promising in vitro and in vivo, the implementation of engineered cell therapies is still painfully slow. In this light, successful strategies to efficiently modulate the hMSCs’ behavior for clinical use are mandatory [33].

As a rule of thumb, an effective gene delivery strategy must be very efficient in transferring nucleic acids (NAs) to target cell populations allowing tighter control of gene expression over time while minimizing cytotoxicity and safety concerns arising from the overall process. This aspect holds particular importance when working with primary cell cultures, such as hMSCs, as they are known to be challenging to transfect. Indeed, these cells exhibit higher sensitivity and lower division rates compared to cell lines, making the successful delivery of genetic material more difficult [34]. So far, much effort has been devoted to devising suitable means to improve the delivery efficiency of NAs into target hMSCs. Such strategies are generally classified into two main categories based on the way used to transfer the genetic cargo into cells: gene delivery through physical methods and delivery mediated by vectors [35].

Generally, physical methods, including electroporation [36–38], sonoporation [39–41], magnetofection [42–44], and micro-injection [45–47], involve the application of mechanical or electrical forces to temporarily create pores in the lipid bilayer of the plasma membrane. This allows for the introduction of the (naked) NA payload. Therefore, physical methods are ideally suited for treating cells cultured as two-dimensional monolayers ex vivo. Overall, these methods are very effective in transferring genetic cargo to hMSCs, as they force the entry of NAs into cells through the transient disruption of the plasma membrane, instead of relying on endocytosis pathways [48]. Nonetheless, side effects related to high cytotoxicity make them somewhat inconvenient [49].

Another popular way to deliver NAs into target cells involves the use of vector-based delivery systems, commonly referred to as carriers. Their function consists in encapsulating and protecting the NAs within particles, significantly improving their delivery to target cells. These carriers can be classified into two main types: viral and non-viral vectors. Among them, viral vectors, that is, engineered viruses completely void of parent virus genes, harness the viral infection pathway while avoiding the subsequent viral protein expression upon transduction of host cells [50]. Although high transduction efficiency and stable gene expression may be achieved with viral carriers, they are still plagued by inherent issues such as the limited size of NAs that can be packed and delivered, random recombination (i.e., oncogenic potential), tropism, cytotoxicity, and immunogenicity [51]. These concerns have prompted the development of non-viral alternatives, providing scalable, robust, and cost-effective solutions.

Within the last few years, non-viral gene delivery vectors are witnessing a surge of interest, which has led to the recent development of effective products such as the Pfizer/BioNTech and Moderna COVID-19 vaccines [52]. The success of COVID-19 vaccines has given new momentum and legitimized the use of non-viral gene delivery strategies in clinics. Non-viral gene delivery vectors encompass various options, including cationic lipids (CLs) and polymers (CPs), which are able to spontaneously interact with NAs, forming complexes known as lipoplexes and polyplexes, respectively. These complexes serve as a vehicle to carry the genetic cargo into host cells [35, 53] through a process called transfection. In addition to CLs and CPs, there are other non-viral gene delivery carriers available, such as inorganic nanoparticles. Examples include calcium phosphate and mesoporous silica nanoparticles, which have demonstrated successful use in gene delivery (for a comprehensive review on this topic, please refer to [54, 55]).

Non-viral vectors have been extensively utilized in vitro over the past two decades to transfer target genes into hMSCs and control their behavior [31, 56] with some success. There is still a long way to go to find an effective way to engineer hMSCs *via* gene transfer. This is mainly due to the general lack of standardized test procedures for the screening and implementation of gene delivery strategies, thus largely contributing to the number of inconsistent findings. The variability in test protocols used to transfer NAs in vitro into patient-derived hMSCs using non-viral vectors has given rise to a very crowded and controversial body of literature that makes it hard to find the most suitable combination of factors and parameters to effectively transfect target cells.

In this scenario, the goal of this review is twofold. On one hand, it provides an overview of the current non-viral vectors and strategies used to deliver NAs to hMSCs in vitro to commit them toward bone and cartilage lineages. On the other hand, we sought to highlight the main protocols and parameters affecting the transfection of hMSCs that should be taken into account when performing in vitro research intended for osteochondral applications and to suggest novel ways to expedite and improve the gene transfer to hMSCs. In such a way, as a proof of concept, we conducted a series of transfection experiments using a 25 kDa branched polyethyleneimine (*b*PEI) as a transfection agent for a plasmid DNA (pDNA) encoding the luciferase reporter gene in primary cultures of MSCs. The purpose of these experiments was to provide the reader with valuable insights into the key factors affecting transfection efficacy in MSCs, including cell density, NA dose, and transfection duration. Besides, this literature survey is intended to point out the most prominent factors and parameters that may affect the transfection output and to provide tips on how to evaluate qualitatively and quantitatively the transfection outcomes in this cell type.

### Non-viral gene delivery carriers for MSCs

Non-viral vectors offer distinct advantages over both viral counterparts and physical methods. Above all, they are renowned for their ease of use, along with the possibility to fine-tune their physicochemical properties. To design complexes that best fit the target hMSCs, a wide variety of polymers and lipids have been devised and optimized over years. Nevertheless, each has *pros* and *cons*, such that the ideal vector has not been identified yet. Herein, we seek to highlight the most prominent non-viral carriers and the progress made in improving their chemical features to achieve great gene transfer to hMSCs without excessive side effects.

➣ Cationic polymers (CPs)

**Fig. 1:**
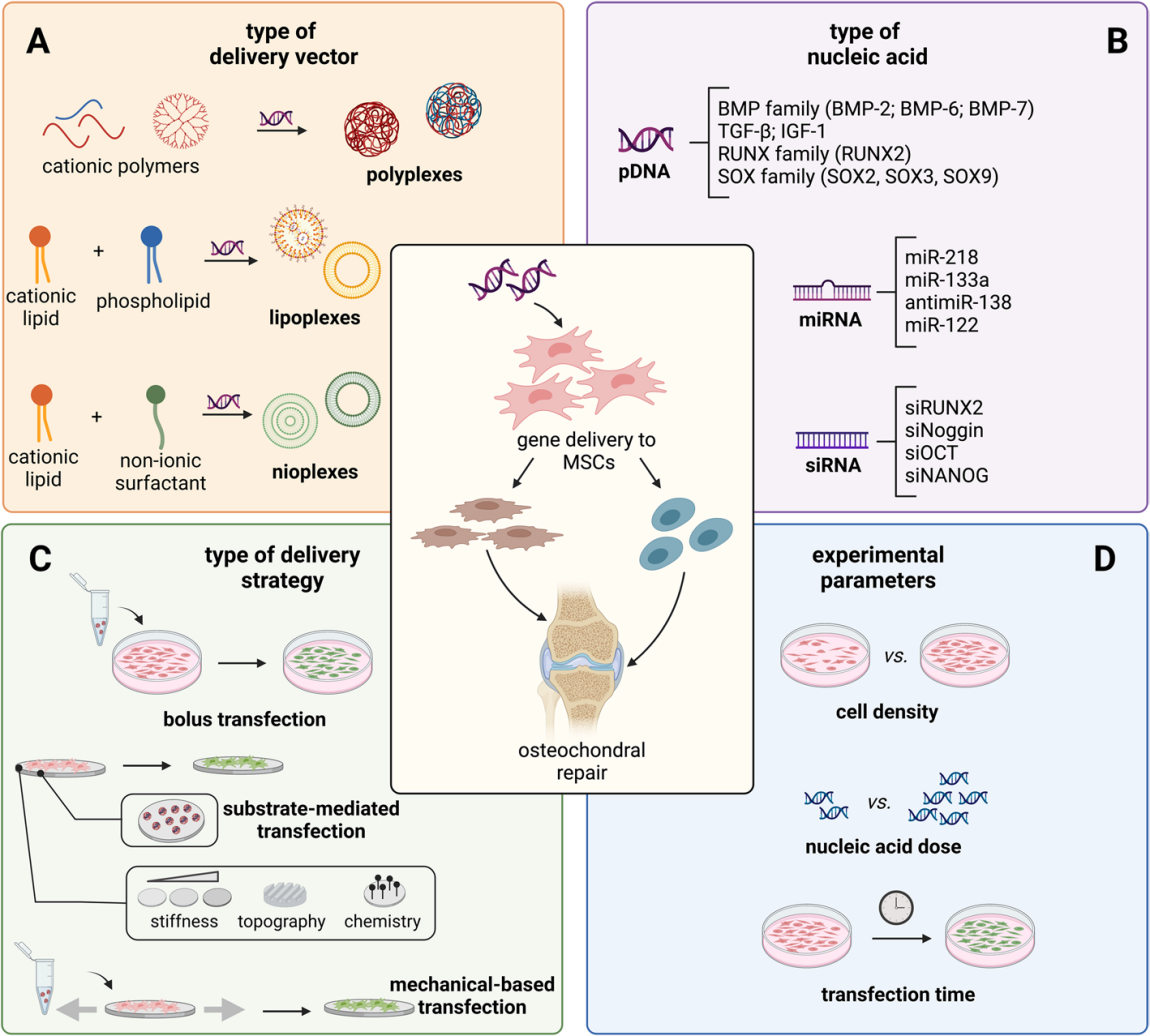
Schematic of the variables and factors affecting non-viral gene delivery to MSCs

Cationic polymers (CPs) (Fig. 1A) represent the first choice when dealing with the transfection of MSCs. Most of the strategies developed so far devised the use of *gold standard* polymeric vectors, namely the class of synthetic polyethyleneimines (PEIs) [23, 27, 28, 57–72], poly-L-lysines (PLLs) [73], and dendrimers such as polyamidoamines (PAMAMs) [74–76], as well as biopolymers such as chitosans [57, 77]. These macromolecules have some protonatable moieties, such as primary, secondary, and tertiary amines, which account for their interaction with NAs and the cell membrane. The advantages of polymer-based carriers rely on their ease of use and the possibility of fine-tuning some features, such as the molecular weight (M_w_), branching degree, and chain length, to achieve a good trade-off between efficiency and inherent cytotoxicity of the resulting complexes [35, 78]. Nonetheless, the performances of CPs are affected by their limited ability to overcome plasma membranes and release NAs intracellularly. Part of these issues has been tackled with some success, such that some promising vectors are in the limelight [73, 79–81].

Different research groups sought to improve effectiveness while reducing the toxicity of some CPs by adding hydrophobic moieties to the polymer. For instance, polyplexes made with PAMAM dendrimers functionalized with hydrocarbon chains of variable lengths showed improved cellular uptake and reduced cytotoxicity if compared to *gold standard* vectors such as 25 kDa *b*PEI [82–84]. Likewise, Uludağ’s group devised PLL functionalized with palmitic acid and PEI with linoleic acid to increase their hydrophobicity. The resulting particles reached the nuclear region more effectively than those obtained with *gold standard* transfection agents, such as Lipofectamine^®^ and 25 kDa *b*PEI, leading to improved effectiveness and decreased cytotoxicity [85, 86].

Other strategies employed to reduce the cytotoxicity of polymer vectors involve the incorporation of biodegradable moieties. These include heterocyclic amines [87], 2,2-bis(hydroxymethyl)propionic acid (bis-MPA) [88], bioreducible disulfide bonds [20, 89, 90], hydrolysable ester bonds [58, 91, 92], β-cyclodextrin (β-CyD) moieties [93, 94]. These modifications have shown significant improvements in transfection efficiency, leading to robust expression levels in MSCs. It is worth noting that, in most cases mentioned above, transgene expression using these smart polymers was enhanced by up to 10 times compared to unmodified PEI, and approximately 2 to 5 times when compared to commercially sourced reagent like Lipofectamine 2000^®^. Therefore, these promising candidates offer a safe and effective means of delivering NAs to MSCs.

More recently, much effort has been devoted to designing non-viral gene delivery vectors featuring targeting ability. To this aim, cell-selective ligands, typically short oligopeptides that bind to cognate cell-surface receptors displayed by target cell populations, have been covalently tethered to polymeric carriers. Taking MSCs as a target, there is a vast literature on this subject. The most thoroughly investigated is the popular RGD tripeptide (arginine-glycine-aspartic acid), which has been used to improve the interaction between the non-viral vectors and the MSCs [81, 95]. Indeed, some transmembrane receptors, such as α_V_β_1_, α_V_β_3_, and α_V_β_5_ integrins, are known to recognize and bind to the RGD consensus motif [96–98]. On the other hand, because such integrins are rather ubiquitous, the functionalization of polymers with RGD can give rise to off-target effects. Other peptides, which are more selective for MSCs, have thus been grafted to CPs and used in transfection with some success [99, 100]. In addition to targeting ad hoc-designed peptides, natural full-length polymers, such as hyaluronic acid (HA), can be used for this purpose. HA is a natural polysaccharide found in most body tissues that is capable of interacting with the CD44 cell-surface glycoprotein of many cell types [101]. It has been shown that hMSCs recognize HA through the CD44 and CD54 receptors [102–104]. As a result, the conjugation of HA to PEI was found to improve the transfection efficiency of the CP itself on MSCs [80, 89, 102].

➣ Cationic lipids (CLs)

Since the first report by Felgner and colleagues in 1987 [105], the use of CL-based carriers for gene delivery has bloomed. CLs are amphiphilic molecules consisting of three building blocks which are i) a protonatable headgroup connected to at least one ii) hydrophobic moiety through iii) a linker [53]. It is worth noting that the structure of CLs can be intentionally modified to enhance their interaction and delivery capabilities for various types of NAs, including pDNA as well as RNA in the form of small interfering RNA (siRNA) and micro RNA (miRNA) (Fig. 1B) [53, 106]. While highly effective in binding anionic NAs, CLs are frequently used in combination with ionic phospholipids or non-ionic surfactants to give supramolecular arrangements such as liposomes or niosomes (Fig. 1A), respectively, that allow for improving their delivery performances [107–109]. Nevertheless, despite the virtually endless list of vectors available, most gene delivery strategies devised for MSCs of different origins rely on the use of Lipofectamine^®^ [20, 110–120], the *gold standard*, commercially-available lipid transfection reagent consisting of a 3:1 (w/w) mixture of the cationic lipid 2,3-dioleoyloxy-N-[2(sperminecarboxiamido)ethyl[-N,N-dimethyl-1-propaniminium trifluoroacetate (DOSPA) and the zwitterionic 1,2-dioleoyl-sn-glycerol-3-phosphoethanolamine (DOPE) [121]. The success of Lipofectamine^®^ relies upon its ease of use and broad-spectrum activity. Lipofectamine^®^-based lipoplexes proved to efficiently treat in vitro either mouse-derived or human MSCs to express osteogenic and chondrogenic genes [47, 122–126]. Aside from very few exceptions, no CL other than Lipofectamine^®^ has been used to transfect MSCs. In this scenario, there is still room for improvement, and the design of novel lipid-based mixtures with improved efficiency and reduced side effects may open the way for effective hMSCs manipulation [127].

On the other hand, niosomes have recently emerged as a promising alternative for the efficient transfection of MSCs. Broadly speaking, niosomes are self-assembled vesicles (Fig. 1A) consisting of a combination of CLs, non-ionic surfactants, helper lipids, and charge modifiers able to interact with NAs and form nioplexes [109, 128]. Experimental evidence demonstrated that niosomes possess higher stability and reduced cytotoxicity compared to their pure CL counterparts. Moreover, they are cheaper than their liposomal counterparts and easier to prepare [109, 128]. In a work by Pedraz’s group, niosomes composed of the CL 2,3-di(tetradecyloxy)propan-1-amine (DTPA) and the non-ionic surfactant polysorbate 80 were used to complex plasmid DNA (pDNA) and transfect mouse-derived MSCs with BMP-7 encoding gene, thus driving the osteogenic differentiation of MSCs with no detrimental effects [129]. Likewise, our group recently developed an effective strategy to transfect hMSCs by using nioplexes consisting of pDNA and a combination of 1,2-di-o-octadecenyl-3-trimethylammonium propane (DOTMA) as the CL, polysorbate 60 as the non-ionic surfactant and cholesterol as helper lipid [130]. The so-formed nioplexes displayed noticeable transfection efficiency and reduced cytotoxicity compared to Lipofectamine^®^-based lipoplexes. These, together with other works found in the literature [131–133], highlight the suitability of such kinds of materials as promising delivery systems for engineering MSCs.

Overall, the safe and effective delivery of therapeutic NAs stretches to MSCs is a hot topic that still represents a challenge for scientists. In this perspective, rational design approaches and the study of the structure–function relationship (SAR), that is, the intimate interconnection between the vector structure and its biological activity, of these delivery carriers will help elucidate the interplay between their efficiency and cytotoxicity.

### Enviromental parameters affecting gene delivery to MSCs

As a matter of fact, the therapeutic potential of engineered hMSCs strongly depends on the transient or stable expression of the proteins of interest upon transfection [134]. Unfortunately, as hMSCs are hard-to-transfect cells, they are preferably engineered ex vivo and subsequently grafted in vivo [56, 134]. In vitro transfections are usually performed by seeding MSCs in monolayers, which are next challenged with lipoplexes or polyplexes directly added to the cell culture medium (i.e., bolus transfection). Although bolus transfection is the simplest way possible to deliver NAs to the cells, the efficiency of the overall process is hampered by the limited mass transport of complexes, stability issues related to their stay in the extracellular *milieu*, as well as the lack of physiological behavior of MSCs in such oversimplified in vitro culture systems. To overcome such drawbacks, other options such as reverse (or substrate-mediated) transfection strategies are now on the hype (Fig. 1C).

As a rule of thumb, the in vitro culture of MSCs mostly relies upon the use of matrices and scaffolds to mimic the in vivo conditions. In contrast to conventional bolus transfection, reverse transfection allows for a higher amount of NAs available per cell and closer control over the cell behavior through chemical and physical cues of the substrate itself [135]. In reverse transfection, NA-containing nanoparticles are immobilized or spotted on a flat (2D) surface or 3D scaffold by different techniques [59]. Cells are then seeded on top of it or embedded within the scaffold. In this light, with the aim to entice MSCs to internalize gene delivery complexes, improve the transfection efficiency and prolong the transgene expression, scientists sought to optimize the substrate properties and design ever more favorable cell-surface interfaces. Generally, 2D or 3D matrices displaying different stiffness, roughness, topography, and surface chemistry have been used to control the MSCs’ behavior and transfectability [136]. As a matter of fact, substrate stiffness per se has been found to induce the osteogenic or chondrogenic differentiation of MSCs *via* integrin-dependent signaling underpinning the mechano-induced MSCs differentiation [137–139]. Likewise, the substrate stiffness has proven to regulate some key mechanisms enabling the internalization of non-viral vectors, thus impacting their effectiveness as well [140–142].

Substrate topography is another determinant in hMSCs differentiation. Of note, the nanostructure provided by the substrate impact focal adhesion assembly at the cell-surface interface. As a consequence, cytoskeletal rearrangements occur. Because the cytoskeleton links the extracellular matrix (ECM) and the nucleus, it ultimately regulates gene expression and cell phenotype [60, 143–145]. Acting at the cell-membrane level, nanotopographical cues have thus been found to impact the internalization of nanoparticles as well [70, 146–148].

Besides, also the substrates’ surface chemistry plays a role in reverse gene delivery [149]. Segura’s group has extensively refined the physical and chemical cues of the substrate to improve the transfection of MSCs [141]. For instance, when mouse-derived MSCs were cultured on a combination of different ECM components, such as collagen type IV, fibronectin, and laminin, the cells did duplicate faster and display higher transgene expression with respect to cells cultured on uncoated tissue culture plates [150]. By the same token, other approaches took advantage of chemical and physical biomimetic environmental cues to extend the transgene expression in MSCs [141, 151–153].

Altogether, this body of evidence suggests a twofold effect of the substrate features, that is, their synergistic effect on the ultimate transfection efficiency and hMSCs phenotype regulation [31]. For instance, some studies suggest the use of softer substrates with an elastic modulus around 25 kPa to commit hMSCs toward a chondrogenic lineage [154–156]. Likewise, other works reported that stiffer substrates with an elastic modulus equal to a few hundred kPa were able to drive MSCs toward osteoblast-like phenotypes when combined with the delivery of complexes containing pDNA encoding the BMP-2 protein [157].

Another greatly overlooked approach relies on the modulation of cell response and improvement of non-viral gene delivery vector effectiveness through exogenous mechanical cues. Different mechanical stimuli have been found to regulate the gene transfer process by easing some steps of the delivery pathway, namely internalization, cytoplasmic transport, and nuclear import of complexes [136, 158]. Indeed, mechanical loading applied to cells is associated with specific cell responses, such as mechanoregulation of membrane trafficking [159–161] and cytoskeletal remodeling [162–167]. In this scenario, cyclic stretch [168–170], shear stress [61, 171, 172], and vibrational loading [62] may boost the transfection efficiency of non-viral gene delivery vectors by enhancing their uptake and intracellular trafficking. Even more interestingly, nanoscale vibrational loading alone has been used to trigger the osteogenic differentiation of hMSCs [63, 173].

Overall, this evidence suggests that the environmental cues to which hMSCs are exposed affect their phenotype, and thus their ability to internalize non-viral particles. Therefore, the use of exogenous stimuli, such as the one exerted by either the substrate or from mechanical loading, coupled with the non-viral gene delivery could represent a promising strategy to develop engineered MSCs with therapeutic potential.

Nevertheless, since there is no advancement without robust screening protocols, the following sections unveil how to harness the most relevant experimental features to improve the efficiency of a given transfectant with MSCs and highlight ways to analyze their efficiency in vitro.

### In vitro transfection assays

#### How to set main transfection parameters

The efficiency of non-viral gene delivery strategies is highly dependent on several factors and conditions used to carry out transfections. Consequently, the optimization of the experimental conditions, such as the cell type and passage number, the cell density, the dose and type of NA to be transferred (Fig. 1B), the type of transfection reagent, and the transfection time are extremely relevant (Fig. 1D).

Broadly speaking, the susceptibility to transfection depends on the cell source, as MSCs can be isolated from different tissues, such as the bone marrow [1, 5, 17, 19, 27, 28, 34, 64–67, 113–117, 120, 132, 135, 141, 151, 152, 174–186] and the adipose tissue [34, 118, 187, 188], and from diverse sources such as human [1, 26–28, 34, 66–68, 86, 110, 112, 115, 119, 152, 153, 174, 176, 177, 181, 183, 187, 189–194], rat [5, 34, 64, 113, 116, 117, 120, 135, 151, 175, 178, 180, 184, 195], murine [20, 34, 118, 132, 141, 181, 182, 185, 196, 197], goat [34, 65, 188], porcine [29, 114, 179], rabbit [186], horse [198], and canine [198]. Therefore, because of the differences in the overall cell metabolism among MSCs coming from different tissues and species, it is difficult to compare the transfection outcomes found in the literature.

Another pivotal parameter affecting transfection outcomes is the cell passage. As a general rule, once extracted from the donor, MSCs are typically transfected between passages 1-to-8 to avoid unintended cell differentiation and aging [20, 26, 34, 65–67, 114, 151, 152, 174, 178, 181–183, 190, 192, 198]. Nevertheless, for achieving more reliable transfection values, it is recommended to avoid cell passages higher than 4.

Another crucial factor influencing transfection efficiency is the type of NA to be delivered. In the case of pDNA, it needs to penetrate the cell nucleus to be functional and express the desired protein. This process occurs either through the disassembly of the nuclear envelope in mitotic cells or *via* pore complexes in non-dividing cells [199]. In contrast, mRNA only requires the cytosolic transcriptional machinery to express the protein product, leading to higher transfection efficiencies compared to pDNA. However, the use of mRNAs entails higher immunogenicity and shorter duration of protein expression compared to pDNA [200]. On the other hand, siRNA and miRNA inhibit the expression of complementary RNAs, thereby modulating cell phenotype and achieving longer duration of expression compared to mRNA [31].

When performing in vitro transfections, both the cell density and the NA dose used to transfect cells significantly impact the gene transfer efficiency as this corresponds to the amount of NAs available on a per-cell basis.

**Fig. 2:**
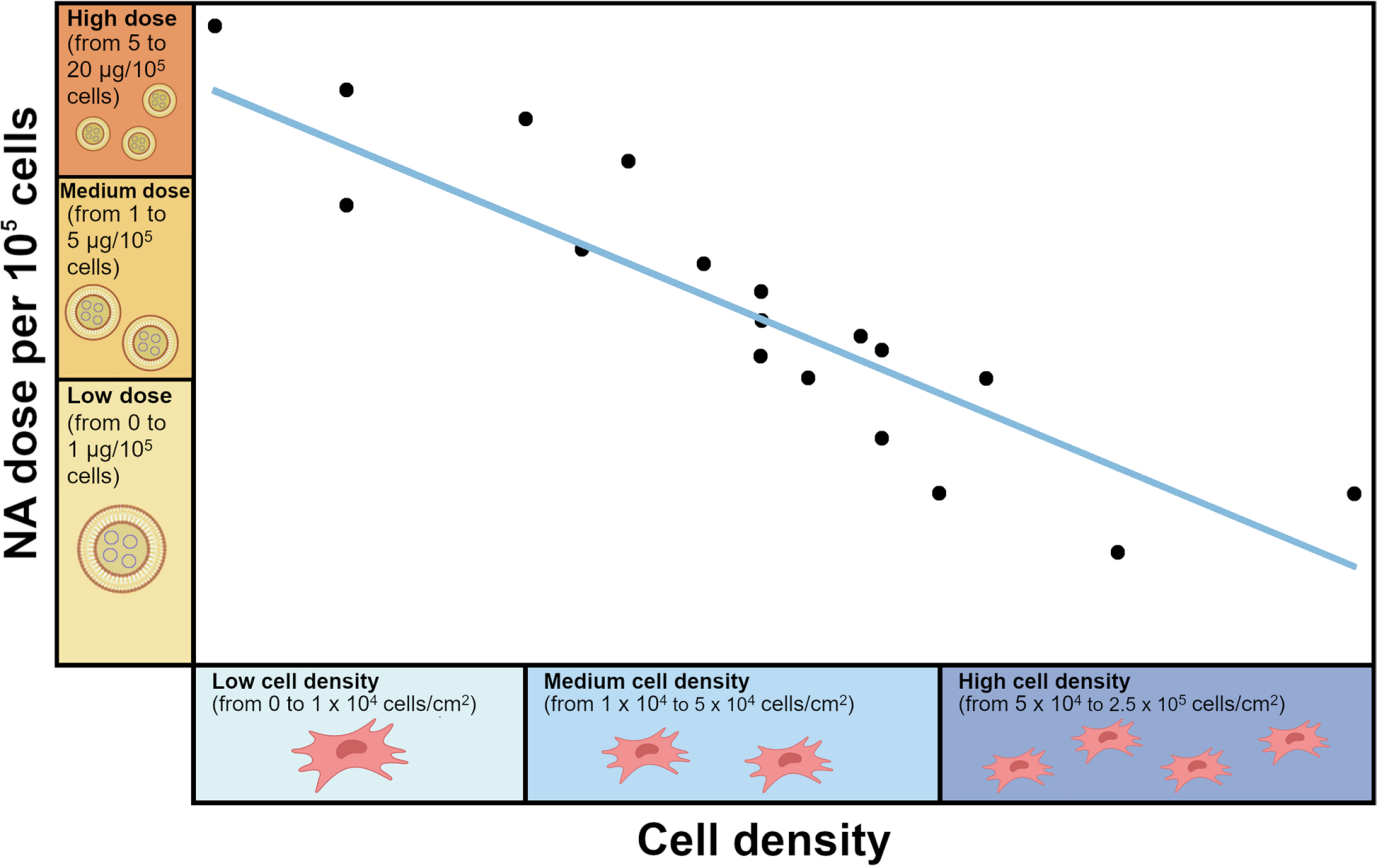
Schematic representation of the correlation between the dose of NAs per 10^5^ cells, ranging from low (from 0 to 1 µg/10^5^ cells), medium (from 1 to 5 µg/10^5^ cells), and high dose (from 5 to 20 µg/10^5^ cells), and the cell density, ranging from low (from 0 to 1 × 10^4^ cells/cm^2^), medium (from 1 to 5 × 10^4^ cells/cm^2^), and high cell density (from 5 × 10^4^ to 2.5 × 10^5^ cells/cm^2^). The trend line (R^2^ = 0.79) represents the tendency of the data taken from articles (references [17, 19, 21, 69, 86, 110, 112, 113, 152, 153, 174–176, 178, 179, 187, 198, 201–203]), where the cell density (cells/cm^2^) and the dose (µg) are specified

Even little variations in a single parameter may give significantly different transfection outcomes, such that a proper trade-off between the NA dose delivered to cells and the cell density should be investigated anytime. To this purpose, a thorough survey of the literature employing non-viral vectors such as PEI [69, 86, 151, 174, 177, 179, 201, 202], Lipofectamine^®^ [198], nano-hydroxyapatite (nHA) [19, 69, 152, 153, 175, 178], linear bioreducible poly(urethane amine) (SSPUA) [187], TransIT [21], and the multi-domain cell-penetrating peptide GAG-binding enhanced transduction (GET) [178], pointed out three main different subsets of MSCs densities used in transfection experiments, such as low (< 1 × 10^4^ cells/cm^2^), medium (from 1 × 10^4^ to 5 × 10^4^ cells/cm^2^), and high cell density (from 5 × 10^4^ to 2.5 × 10^5^ cells/cm^2^). The same applies to the NA dose delivered to cells. In the latter case, experiments can be grouped into low (< 0.5 µg/cm^2^), medium (from 0.5 to 1.5 µg/cm^2^), and high NA dose (from 1.5 to 3 µg/cm^2^). Unfortunately, no exclusive relationship between these two parameters (cell density/cm^2^ and NA µg/cm^2^) can be established since they were not proportionally scaled in the different studies. Therefore, in order to compare the variety of conditions used by different authors, we normalized the NA dose as µg of plasmid per 10^5^ cells and we found a linear fitting between cell densities and plasmid doses, falling most of the studies conditions in the intermedium range (Fig. 2).

Nonetheless, due to the variation in cell densities, NA doses and methods used to analyse transfection, it is not possible to make definitive conclusions regarding transfection efficiency. To shed more light on this issue, we herein provide some genuine results on MSCs and draw some conclusions on the interplay among the cell density, the NA dose, and the transfection time. Transfection studies were thus carried out by using the benchmark transfectant 25 kDa *b*PEI to complex the pDNA encoding the luciferase reporter gene *luc2* (hereafter referred to as p*luc*) at the extremely effective cationic polymer amino groups (N) to the anionic NA phosphate moieties (P) ratio (N/P) of 10 [152, 174].

**Fig. 3:**
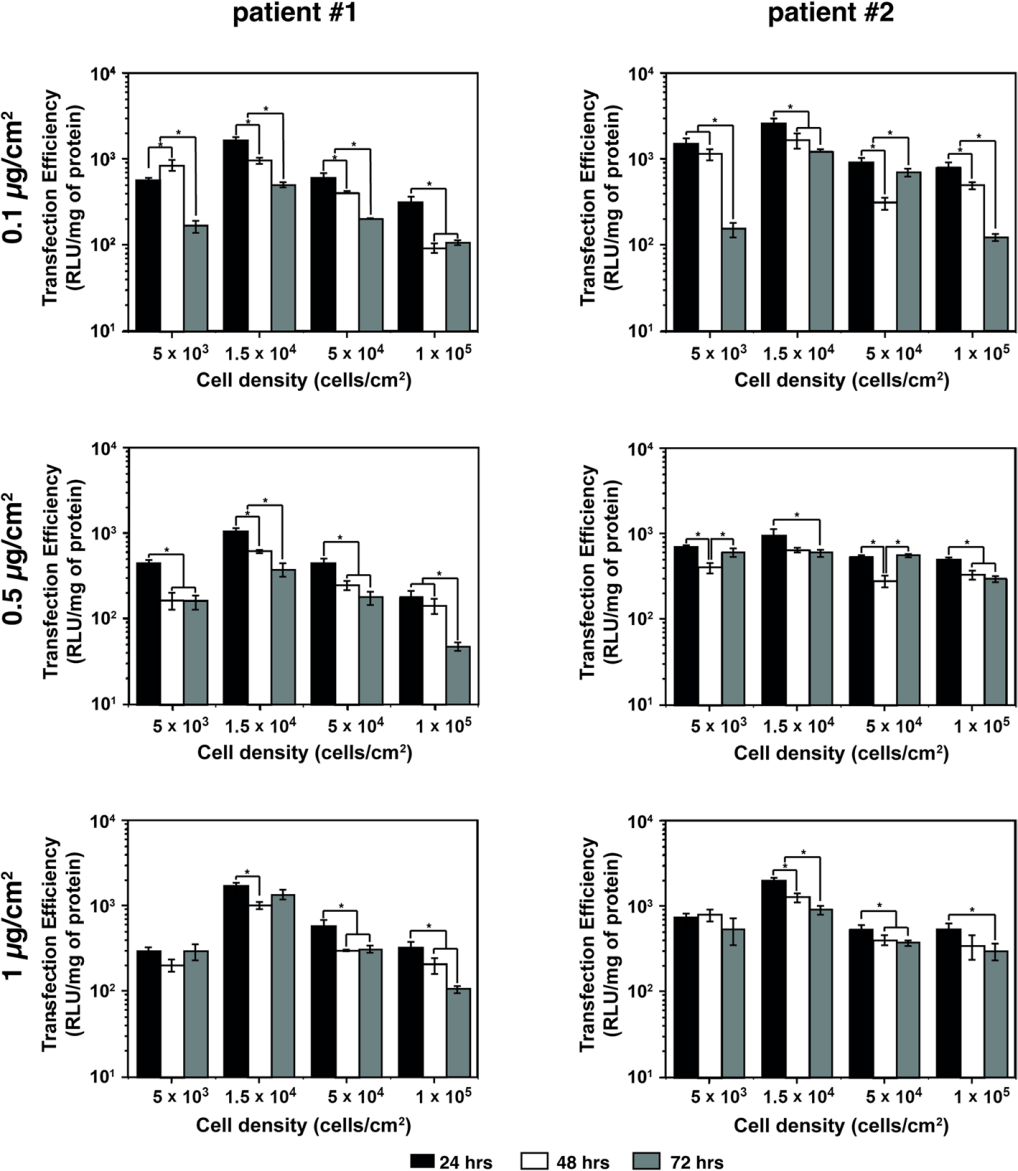
Transfection efficiency, expressed as luciferase activity normalized to the total protein content (RLU/mg of protein), following transfection with *b*PEI/p*luc* complexes prepared at N/P 10 on hMSCs isolated from two patients (i.e., patient #1 and patient #2, right and left panels, respectively) as a function of the pDNA dose (0.1, 0.5, and 1 µg/cm^2^) and the cell density (5 × 10^3^, 1.5 × 10^4^, 5 × 10^4^, and 1 × 10^5^ cells/cm^2^). Luciferase activity was evaluated 24, 48, and 72 h post-transfection. Results are expressed as mean ± SD (*n* = 3, * *p* < 0.05)

Transfections were carried out on bone marrow-derived-MSCs from two patients herein named #1 and #2, kept in culture, and transfected between passages 1 and 2. The transgene expression was evaluated 24, 48, and 72 h post-addition of polyplexes to the cells (Fig. 3).

In light of the results depicted in Fig. 3, we draw some conclusions on the role of the cell density and pDNA dose on the ultimate transfection efficiency of *b*PEI-based polyplexes and propose a guideline on how to design in vitro assays for MSCs’ transfection. Interestingly, regardless of the pDNA dose used, the cell seeding density of 1.5 × 10^4^ cells/cm^2^ led to the highest levels of luciferase activity (up to a maximum 12.8-fold difference compared to any other cell density tested; *p* < 0.003). Therefore, we assume that the optimal MSCs density to carry out transfection assays should be set close to this value. It is worthy of note that transgene expression strongly depends on cell doubling [35], in the way that exogenous pDNA freely enters nuclei due to the nuclear cell membrane disruption occurring during mitotic events. Based on our results, we can speculate that cells seeded at a density of 1.5 × 10^4^ cells/cm^2^ are in a high proliferative state, such that more pDNA is made available to the transcription machinery.

Since most of the studies employ transfection time windows, i.e., the time elapsing between the delivery of complexes to the cells in culture and the transgene expression analysis, between 24 and 72 h [20, 21, 26, 65, 68, 110, 118, 132, 177, 178, 182, 184, 185, 190, 204], or even longer [29, 181, 186], we sought also to elucidate the effect of this parameter on the transgene expression. Interestingly, irrespective of the cell density, the highest luciferase activity was reached 24 h-post polyplex delivery (up to a 10.0-fold difference compared to any other time point; *p* < 0.001). This is an interesting point since it means that maximum transgene expression of the luciferase gene occurs in this timeframe regardless of the cell density, NA dose, and cell source used. However, we cannot generalize the conclusion to every transgene of interest as each has specific expression patterns. This implies that good experimental practice should include the optimization of the transfection time depending on the transgene used.

Besides, the results reported in Fig. 3 suggest that the pDNA dose used to transfect cells has, to some extent, no significant influence on the effectiveness of *b*PEI-polyplexes and can be varied as long as it does not affect the viability of hMSCs, which was > 85% in any conditions (Figure S1).

### How to evaluate the distribution and internalization pathways of complexes into transfected MSCs

To be effective, non-viral gene delivery vectors must be able to overcome many extra- and intracellular barriers, that is, rate-limiting steps which include the crossing of the cell membrane, the release from endo-lysosomes in the cytoplasm, and the nuclear import of NAs (when the delivery involves DNA). Therefore, the uptake and intracellular trafficking of complexes should be assessed especially when screening new reagents.

Overall, shedding light on the structure–activity relationship of the vector will allow the identification and optimization of essential parameters, such as the shape, size, and surface charge of nanoparticles, that are crucial for efficient gene delivery [205].

As for the cell uptake, protocols commonly used to assess the internalization and intracellular distribution of the complexes rely on the use of fluorescently labeled NAs. As summarized in Table 1, most works exploit cyanine dyes, as they cover a wide range of wavelengths depending on the length of the polyalkene bridge connecting the two nitrogen heterocyclics, hence providing versatility for diverse applications [19, 27, 66, 86, 116–118, 184]. Compared to other traditional dyes such as fluorescein [5, 27] and rhodamine [113, 189], cyanines exhibit enhanced water solubility and photostability, being less sensitive to pH changes, *i.e.* those occurring when complexes enter the endo-lysosomal compartment. Other molecules used to examine complexes uptake include fluorophores such as Dy547 [34], YOYO-1 [177, 187], TAMRA [185], and Texas Red [174]. While Dy547 and YOYO-1 increase their fluorescence once bound to NAs, TAMRA and Texas Red can be used to label either the vector, the NAs, or both of them, and allow acquiring further insight into the internalization process.

**Table 1:** Current strategies to evaluate internalization of complexes into MSCs including uptake and inhibition assays

NAs	Fluorescent probe for labeling	Endocytic inhibitors	Analytical methods	Ref
miRNA (miR-218)	Alexa Fluor 647 (red, miRNA), Lysotracker Green (green, lysosomes), and Hoechst 33342 (UV, cell nucleus)	NO	Confocal microscopy	[182]
pDNA (pBMP-2)	FITC (green, PEI) and DAPI (UV, cell nucleus)	NO	Confocal microscopy	[5]
pDNA (pGFP)	Cy3 (red, pDNA), WGA-Alexa Fluor (green, cytoplasm) and DAPI (UV, cell nucleus)	NO	Cy3 positive by FCM and confocal microscopy	[86]
miRNA (miR-133a)	Dy547 (red, miRNA)	NO	Fluorescence plate reader	[206]
pDNA (pBMP-2)	YOYO-1 (red, pDNA) and DAPI (UV, cell nucleus)	NO	Confocal microscopy	[177]
pDNA (pGFP and p*luc*)	YOYO-1 (red, pDNA)	Chlorpromazine, wortmannin, genistein, Me-β-CD, baf-A1, nocodazol, and aphidicolin	Confocal microscopy (internalization) and luminescence plate reader (p*luc* inhibition)	[187]
pDNA (pBMP-2)	Alex Fluor 594 conjugated dextran (red, MCMs) and pGFP (green)	Chlorpromazine, Me-β-CD, and amiloride	Fluorescence microscopy (internalization and pGFP inhibition)	[190]
pDNA (pBMP-2)	NO	Sucrose, LY294002, Me-β-CD and amiloride	ELISA (pBMP-2 inhibition)	[110]
siRNA (siRUNX2)	TAMRA (red, siRNA)	NO	TAMRA positive by FCM and fluorescence microscopy	[185]
pDNA (pBMP-2 and pTGF-β3)	Cy3 (red, pDNA), Alexa 488 Phalloidin (green, actin filaments) and DAPI (UV, cell nucleus)	NO	Confocal microscopy	[19]
miRNA (antmiR-138)	Cy3 (red, miRNA) and DAPI (UV, cell nucleus)	NO	Fluorescence microscopy	[184]
siRNA (siNoggin)	Cy3 (red, siRNA)	NO	Fluorescence microscopy	[118]
pDNA (pGFP and p*luc*)	Rhodamine B (red, peptide) and Hoechst 33342 (UV, cell nucleus)	NO	Fluorescence microscopy	[189]
pDNA (pGFP)	Rhodamine B (red, polyamide), Lysotracker Green DND-26 (green, endosomes) and DAPI (UV, cell nucleus)	Chlorpromazine, genistein, Me-β-CD, and amiloride	Confocal microscopy (internalization) and FCM (pGFP inhibition)	[113]
pDNA (pOSX-GFP)	Texas red (red, PEI) and DAPI (UV, cell nucleus)	NO	Confocal microscopy	[174]
miRNA	Dy547 (red, miRNA) and Calcein-AM (green, cytoplasm)	NO	Fluorescence microscopy	[207]
miRNA (antmiR-138)	Cy3 (red, miRNA) and Hoechst 33342 (UV, cell nucleus)	NO	Fluorescence microscopy	[117]
miRNA (miR335-5p)	Cy5 (red, pDNA), Atto 565 dye (pink-red, MNPs), FluoReporter Oregon Green^®^ (green, PEI), and DAPI (UV, cell nucleus)	NO	Confocal microscopy	[66]
miRNA (antmiR-138)	Cy3 (red, miRNA) and DIO (green, cell membrane)	NO	Confocal microscopy	[116]
pDNA (pTGF-β1)	NO	Chlorpromazine	Luminescence plate reader (p*luc*)	[135]
pDNA (p*sox9*)	Cy5 (red, pDNA), RITC (red, NPs) and FITC (green, PEI-PLGA nanoparticles)	NO	Confocal microscopy	[27]

Abbreviations: *miRNA* micro-RNA, *UV* ultraviolet, *pDNA* plasmid DNA, *BMP-2* bone morphogenic protein-2, *FITC* fluorescein isothiocyanate, *PEI* polyethyleneimine, *DAPI* 4', 6-diamidino-2-phenylindol, *OSX* osterix, *GFP* green fluorescent protein, *Cy3* cyanine 3, *luc* luciferase, *Me-β-CD* methyl-β-cyclodextrin, *baf-A1* bafilomycin A1, *MCM* mineral-coated microparticles, *RUNX2* runt-related transcription factor 2, *Cy5* cyanine 5, *TGF-β1 and -β3* transforming growth factor-β1 and -β3, *sox9* sex-determining region Y-type high mobility group box 9, *RITC* rhodamine isothiocyanate, *NPs* nanoparticles, *PLGA* poly (DL-lactic-co-glycolic acid)

Flow cytometry (FCM) is by far the most effective method to gather quantitative data on complex internalization by cells. To this aim, fluorescence-labeled gene delivery complexes are added to cell monolayers and incubated for a certain time. At given time points, cells are harvested, fixed, the fluorescent signal is measured using an FCM and compared to negative controls (i.e., untransfected cells) [27, 34, 66, 86, 116–118]. Alternatively, confocal microscopy (CSM) allows one to get insights into the in-cell distribution of the labeled complexes. Briefly, following transfection, cells are fixed, stained, and mounted on cover slides before being imaged using a fluorescent microscope. While the staining protocol depends on the specific targets, the most used procedures make use of nuclear dyes (e.g., 4,6-diamino-2-phenylindole—DAPI, Hoechst 34580, Hoechst 33258, and Hoechst 33342 that bind to DNA) and cytoplasmic stains (specific antibodies, e.g., anti-tubulin, or binders, e.g., phalloidin, coupled to fluorochromes, such as Alexa Fluor) [5, 19, 34, 184, 185, 187].

Most studies are performed to shed more light on the complexes uptake and internalization pathways. The endocytic routes can be thus selectively inhibited to assess the contribution of each pathway to the particle uptake. The inhibitors currently used in internalization assays comprise *i)* chlorpromazine to block clathrin-mediated endocytosis (CME) [113, 135, 187, 190], *ii)* genistein, filipin, or methyl-β-cyclodextrin to inhibit caveolae-mediated endocytosis (CvME) [110, 113, 187, 190] and *iii)* wortmannin [113, 187], or amiloride [110, 190] to suppress macropinocytosis, that is, the three main entry routes for non-viral gene delivery vectors [208]. It has been shown that particle size plays an essential role in the internalization pathway, and most particles with a diameter ranging from 50 to 500 nm are internalized through CME or CvME pathways. However, while CL-based particles are typically internalized *via* CME, polyplexes are usually internalized through both CME and CvME pathways [72]. To elucidate the role of each endocytic route on complexes uptake and transfection efficiency, cells are incubated with specific endocytosis inhibitors before carrying out transfection. The ultimate expression of a reporter gene (e.g., Green Fluorescent Protein (GFP) [113, 187, 190], luciferase (*luc*) [135]) or therapeutic transgene (e.g., BMP-2 [110]) is analyzed afterward and compared to that of cells transfected in the absence of such inhibitors.

Regardless of the internalization route, lipoplexes and polyplexes end up in vesicles called endosomes, which are another intracellular barrier toward effective gene transfer. Tracking the intracellular journey of complexes through the staining of the lysosomal compartment can help elucidate the intracellular fate of nanoparticles. The information gained can be used to design endosomal escape strategies. To improve transfection, some authors exploited the use of media additives, *i.e.*, molecules exerting an effect by enhancing the transfection efficiency in MSCs. For instance, Pannier’s group introduced the use of glucocorticoids, such as dexamethasone (DEX), to improve transgene expression in MSCs. This strategy has proven successful for various NAs and has paved the way for its application in numerous fields. Additionally, DEX has shown pro-anabolic effects in MSCs chondrogenesis and is commonly used in the formulation of chondrogenic cell culture media. Specifically, the use of glucocorticoids was found to positively counteract the cytotoxic effect of non-viral vectors by decreasing cell-oxidative stress arising as a consequence of complexes’ delivery and preventing the decline of cell metabolism [209, 210]. Likewise, our group devised the use of sucrose as a lysosomotropic agent to enhance complexes escape from the endo-lysosomes, thus favoring their cytoplasmatic transport [211]. Briefly, MSCs were transfected with nioplexes made of DOTMA, polysorbate 60 and cholesterol, and complexing pDNA encoding the β-galactosidase reporter in a sucrose-enriched (i.e., 40 mM sucrose) culture medium. As a result, the transgene expression improved due to the rise in intracellular pDNA content [130], whereas cell viability was not affected.

A thorough understanding of the uptake and intracellular trafficking pathways of complexes is needed when dealing with new non-viral vectors. This issue becomes even more relevant when using hard-to-transfect cells, such as hMSCs. The combination of different experimental tools, such as fluorescence microscopy, FCM, and the use of endocytic inhibitors thus represents a straightforward way to design ever more effective delivery strategies to engineer hMSCs.

### How to evaluate cell viability and transfection efficiency following transfection

**Fig. 4:**
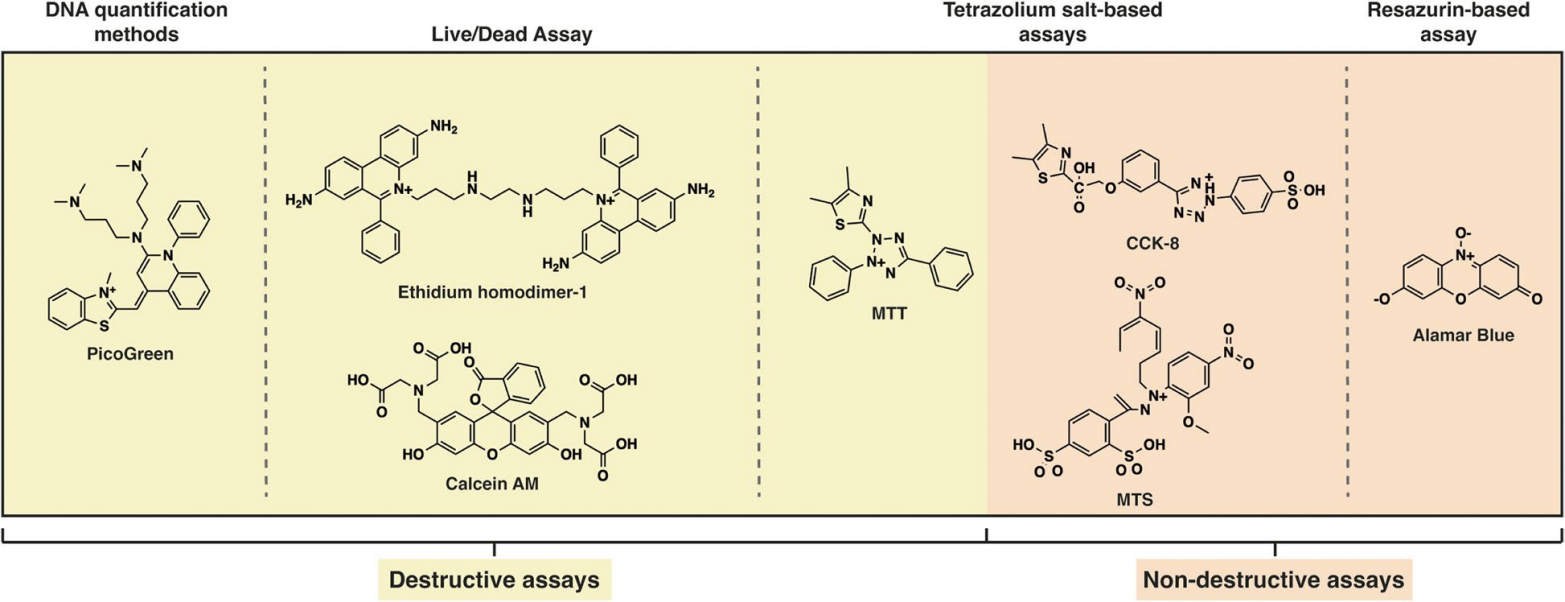
Common colorimetric indicators used to evaluate the cytotoxicity of non-viral gene delivery carriers

The assessment of cell viability following transfection allows one to evaluate the cytotoxic effects of non-viral carriers. The oldest methods to assess cell viability comprised the use of dye exclusion assays to determine the number of live and dead cells through microscope analysis and FCM. The most used cell membrane-impermeable dyes are Trypan blue [112, 204], 7-AAD (7-aminoactinomycin D) [112], and propidium iodide (PI) [133, 212]. Novel and simpler viability assays based on the use of biochemical markers are now preferred (and preferable) to evaluate cell metabolic activity. Most common cell viability assays include MTT [17, 26, 27, 86, 110, 135, 141, 152, 178, 182, 185, 189, 192, 194], MTS [5, 34, 67, 68, 153, 176, 177], CCK-8 [20, 21, 34, 116, 184], Live/Dead assay [5, 19, 29, 67, 132, 141, 174, 175, 185, 191, 193, 197], resazurin assays [34, 113, 119, 179, 187], and DNA-based quantification kits [28, 34, 69, 114, 120, 188, 194] (Fig. 4).

Current methods for cell viability assessment, such as MTT, CCK-8, and MTS, rely on the intracellular reduction of tetrazolium salts into formazan dyes that are measured spectrophotometrically to evaluate viable cells. While the MTT assay requires an additional step to dissolve intracellular water-insoluble formazan crystals, MTS and CCK-8 comprise water-soluble formazan products and allow one to avoid the final solubilization step [213, 214].

Conversely, resazurin-based methods require reduction by cell dehydrogenases using NADH/NADPH as co-substrates. The most commonly resazurin-based methods used are Alamar Blue^®^ [19, 113, 118, 179, 187, 196] and CellTiter-Blue^®^ Cell Viability Assay [34, 119, 190]. In contrast to destructive assays, such as most tetrazolium salt-based assays, the last ones do not even interfere with cell metabolism.

Another assay frequently used to investigate cell viability is Live/Dead staining based on the simultaneous labeling of viable and dead cells by combining a membrane-permeable dye, such as Calcein-AM (green fluorescence), and a membrane-impermeable high-affinity DNA stain, such as ethidium homodimer-1 (red fluorescence) [213].

Besides, the side effects of transfection on cell viability can also be assessed by quantifying the nuclear DNA content in transfected cells. On this note, Quant-it Pico Green dsDNA Kit is one of the most reliable assays used for this purpose [28, 34, 120, 188, 194].

**Fig. 5:**
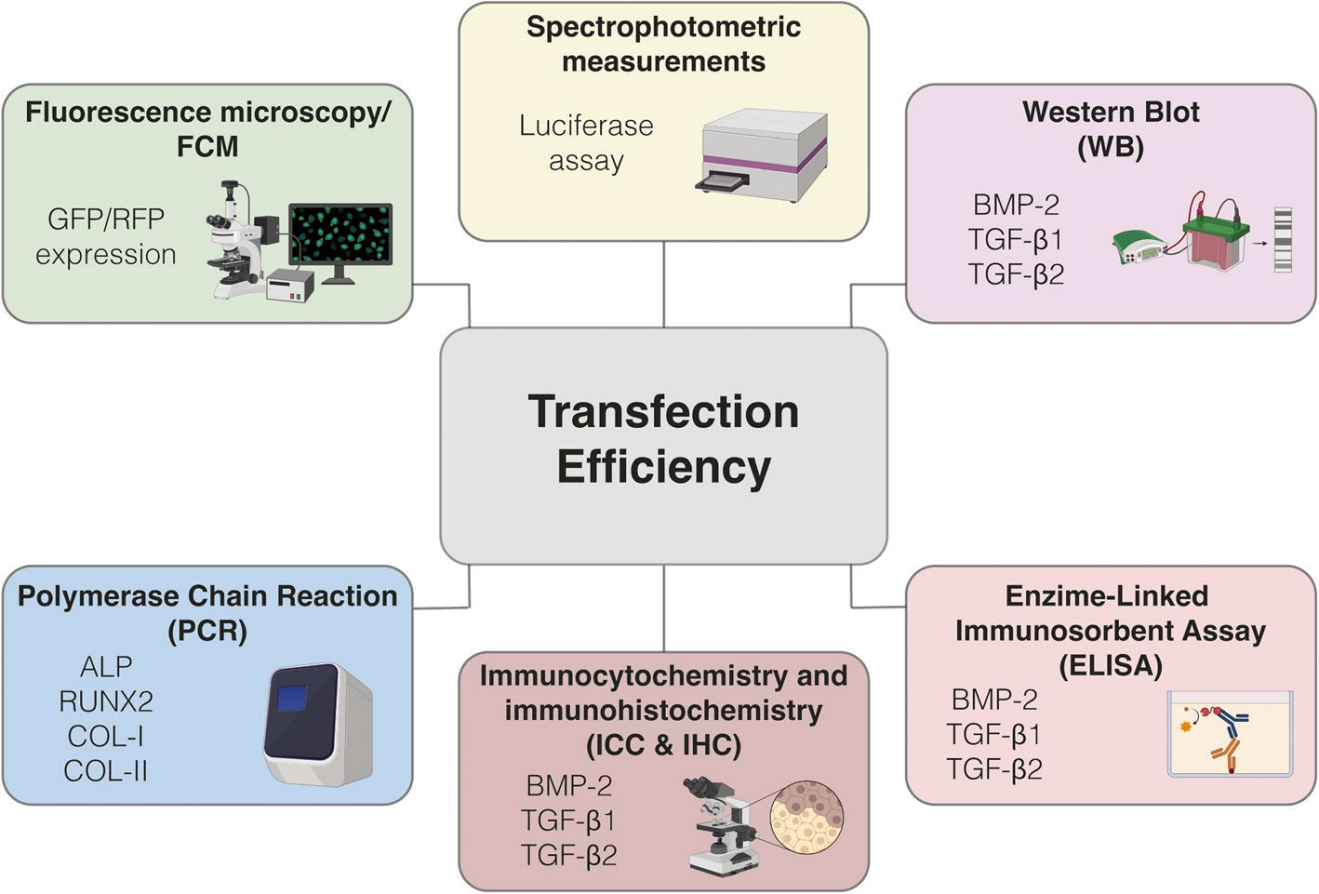
Schematic classification of the most popular methods and technologies used to gauge the transfection efficiency of non-viral gene delivery carriers

Assessing the transfection efficiency is the primary way to gauge the ability of a non-viral gene carrier to introduce exogenous NAs into a specific cell type and alter the expression of a target gene or genes. Depending on the transgene being expressed or the genetic sequence silenced, different methods are available to assess the transfection efficiency of non-viral vectors, including *i)* spectrophotometric measurements, *ii)* fluorescence microscopy, *iii)* FCM, *iv)* polymerase chain reaction (PCR), *v)* enzyme-linked immunosorbent assay (ELISA), Western Blot (WB), and *vi)* immunocyto/immunohistochemical assays (Fig. 5).

On the other hand, the most common screening methods to analyze transfection efficiency are based on the assessment of the activity of the luciferase enzyme (*luc*) encoded by the corresponding reporter gene [35, 96], and fluorescence microscopy and FCM analyses, if the reporter transgene delivered encodes for a fluorescent protein, such as GFP, red fluorescence protein (RFP), and yellow fluorescent protein (YFP) [215]. Luciferase assays rely on the enzymatic conversion of the luciferin substrate into oxyluciferin with concomitant emission of photons (luminescence) by transfected cells [34]. The assay is rapid, sensitive, and specific, as it is compatible with most existing viability tests [216]. Conversely, fluorescence microscopy allows to determine the transfection efficiency through image analysis of transfected *vs.* untransfected cells [217]. This technique is simple yet time-consuming and cumbersome. Hence, FCM allows for discrimination among non-transfected and transfected cells by manually gating the fluorescence signal of a cell subpopulation in a sample, providing the percentage of transfected cells over the total cell number. Of note, this technique is quantitative, sensitive, and enables the fast analysis of large cell numbers [217].

Whereas PCR is a widely used technology for the exponential amplification of a DNA fragment, quantitative PCR (qPCR) is the *gold standard* for the detection and quantification of a NA target. In brief, transfected cells are lysed, mRNAs are recovered, and reverse transcribed through RT-PCR to give a pool of complementary DNAs. Next, the NA stretch of interest is amplified and quantified using qPCR. More often, the NAs delivered in transfection assays are pDNAs encoding for osteo- or chondrogenic factors [18, 26–28, 34, 67, 117, 118, 120, 153, 174, 177, 179–183, 186, 191, 193–197, 206, 218], but it is fairly frequent to use micro-RNAs (miRNAs), that is, a class of noncoding RNAs inhibiting the translation of the mRNA involved in MSCs differentiation [116], such as miR-335 [66], miR26-a [181], miR-138 [117, 184], miR133a [116, 206], miR-218 [182], or miR-122 [180]. Even if qPCR is highly sensitive, it is a destructive technique requiring the disruption of the samples under investigation before analysis. This implies that it is not suited to carry out time-course experiments.

To probe the post-transfection expression of osteo- or chondrogenic markers other than mRNAs, ELISA or WB are routinely performed. ELISA is the most widely used method to unambiguously detect and quantify the concentration of a given protein, which relies on the binding affinity of an antibody-antigen pair [219]. In the case of soluble, secreted markers, cells are transfected and cultured for the desired timeframe. Culture medium aliquots are harvested at fixed times for ELISA analysis to quantify the expression of the target protein over time. Most common examples of proteins assessed by ELISA include BMP-2 [19, 21, 29, 34, 65, 69, 114, 115, 119, 153, 178, 179, 186, 190, 197], TGF-β1 [1, 17, 18, 114, 186], TGF-β3 [19, 153, 179], and vascular endothelial growth factor (VEGF) [69, 178, 181].

On the other hand, WB assay [20, 27, 28, 181, 182] relies on the specific interaction of antibodies with target antigens (osteo- or chondrogenic proteins) present in the culture media or intracellularly. WB allows separating proteins based on size through a molecular sieve (i.e., sodium dodecyl sulfate–polyacrylamide gel electrophoresis (SDS-PAGE)), transferring them onto a solid support, and identifying the protein of interest using specific antibodies labeled with a tag, such as horseradish peroxidase (HRP). WB is cheaper than ELISA on a per-sample basis, but it is a semi-quantitative (image processing), cumbersome, and time-consuming technique.

Other methods to assess the presence of specific proteins in cell monolayers or tissue sections are the immunocytochemical (ICC) and immunohistochemical (IHC) analyses. The main advantage of these techniques lies in the ability to detect transgene-encoded proteins while preserving the structural integrity of the samples, thus enabling the localization of the target antigen(s) in situ. Conversely, IHC and ICC are essentially qualitative. The operating principle is similar to that of WB and ELISA, which means taking advantage of the antibodies to recognize the target proteins and visualize them using chromogenic or fluorescent-based detections. In the former case, the detection is made possible by the enzymatic cleavage of a substrate to give a visible precipitate, whereas, in the latter one, a fluorophore is conjugated to the antibody specific to the target protein, and the signal is detected through fluorescence microscopy.

Standard protocols for IHC require the specimen fixation in paraformaldehyde (4—10% (v/v) in water), followed by its inclusion in paraffin or cryo-embedding media, such as optimal cutting temperature (OCT) compound, and sectioning. For the staining of paraffin-embedded sections, samples required to be first dewaxed in a xylene-ethanol gradient and rehydrated, while OCT-embedded samples required to warm the slides to room temperature to remove the embedding medium. On the other hand, ICC constitute the easiest and practical option, requiring only the fixation of the samples.

In all cases, the samples undergo a series of steps preparatory to the addition and incubation with antibodies and, eventually, the chromogenic substrate. Finally, the mounting medium is added to the slices, which are visualized through microscopy. IHC and ICC are suitable for different applications including the evaluation of the expression of specific transgenes, such as SOX9 [28] or NANOG [26]. To increase the number of details, IHC can be combined with other counterstaining, such as hematoxylin or eosin [1, 111, 114, 117, 176, 180, 183]. This traditional histological technique relies on the affinity of the different cell components for each dye based on their acid–base nature. Therefore, whereas hematoxylin stains in purplish blue basophilic organelles such as nuclei, eosin counterstains in pink the basic cell compartments such as the cytoplasm [5, 18, 23, 34, 69, 114, 118, 135, 153, 186, 188, 190, 206, 218].

### How to induce and evaluate MSC differentiation

The inherent ability of MSCs to differentiate into mesoderm lineage cells, such as osteoblasts and chondrocytes, points out their great potential in osteochondral repair [220]. Despite their promising potential, the direct transplantation or intra-articular injection of MSCs often results in a mixture of hypertrophic, cartilaginous, and fibrous tissues [220]. Consequently, a significant body of research has been dedicated to gene delivery strategies aimed at committing MSCs to express specific proteins and factors, ultimately directing their differentiation toward a desired cell phenotype (Table 2). However, it is worth highlighting that high levels of transgene expression do not necessarily lead to increased MSCs differentiation. Therefore, the selection of an appropriate delivery vector greatly influences the bioactivity of the gene product and the attainment of the desired cell phenotype [179].

**Table 2:** Non-viral gene-delivery based therapeutic approaches for bone and/or cartilage repair

(Therapeutic NA)	Non-viral vector	In vitro / in vivo study	Approach	Methods	Ref
pDNA (BMP-2)	PEI polyplexes	In vitro (BMMSCs)/ In vivo (rabbit model)	Bone regeneration	Histological, IHC, and immunofluorescence analyses	[5]
pDNA (BMP-2 and FGF-2)	PEI polyplexes	In vitro (DPMSCs)/ Ex vivo (tooth model)	Bone regeneration	ELISA, PCR, and ICC analyses	[202]
pDNA (BMP-2)	nHA	In vitro BMMSCs)/ In vivo (mice model)	Bone regeneration	ELISA, histological and IHC analyses	[23]
pDNA (BMP-2)	nHA	In vitro (BMMSCs)/ In vivo (rat model)	Bone regeneration	ELISA, histological and IHC analyses	[22]
pDNA (BMP-2, and VEGF)	GET peptide complexes	In vitro (rMSCs) / In vivo (rat model)	Bone regeneration	ELISA, and histological analyses	[178]
pDNA (BMP-2)	TransIT^®^ lipopolyplexes	In vitro (BMMSCs)/ In vivo (rat model)	Bone regeneration	ELISA, histological and IHC analyses	[21]
pDNA (BMP-2)	Poly (amido amine) polyplexes	In vitro (TDMSCs)/ In vivo (mice model)	Bone regeneration	PCR, WB, histological and IHC analyses	[20]
pDNA (BMP-2/6 and BMP-2/7)	Nucleofector^®^	In vitro (gMSCs)/ In vivo (mice model)	Bone regeneration	ELISA, and histological analysis	[65]
pDNA (BMP-2 and VEGF)	PEI polyplexes and nHA	In vitro (rMSCs) / In vivo (rat model)	Bone regeneration	ELISA, and histological analysis	[69]
pDNA (PDGF B)	PEI polyplexes	In vitro (BMMSCs)/ In vivo (rat model)	Bone regeneration	Histological and IHC analyses	[176]
pDNA (TGF-β1)	Peptide complexes	In vitro (BMMSCs)/ In vivo (rabbit model)	Bone regeneration	ALP assay, PCR, and histological analysis	[18]
pDNA (BMP-2)	PEI polyplexes	In vitro (BMMSCs)	Bone regeneration	PCR, and CC analysis	[177]
pDNA (BMP-2)	nHA (mineral coated)	In vitro (hMSCs)	Bone regeneration	ELISA, and CC analysis	[190]
pDNA (BMP-2/9)	PEI polyplexes	In vitro (BMMSCs) / In vivo (rat model)	Bone regeneration	PCR, histological analysis, and atomic absorption spectroscopy	[218]
PDNA/ (BMP-2 modified)	Peqfect^®^ peptide complexes	In vitro (rAMSCs)	Bone regeneration	ALP assay, ELISA, PCR, and CC analysis	[203]
pDNA (TGF-β1)	Stearate cationic peptide complexes	In vitro (MSCs)	Bone regeneration	ALP assay	[189]
pDNA (OSX-GFP)	*b*PEI polyplexes	In vitro (hBMMSCs)	Bone regeneration	CC analysis	[174]
pDNA (ephrinB2)	*b*PEI polyplexes	In vitro (hBMMSCs)	Bone regeneration	PCR, Calcium Liquicolor kit, and blocking peptide assay	[152]
pDNA (BMP-2)	nHA and Lipofectamine 2000^®^	In vitro (rMSCs)	Bone regeneration	Calcium Liquicolor kit, CC and immunofluorescence analyses	[175]
cDNA (BMP-2)	Lipofectamine 2000^®^ lipoplexes	In vitro (BMMSCs)/ In vivo (mice model)	Bone regeneration	ELISA, and histological analysis	[115]
miRNA (miR-133a)	nHA	In vitro (rMSCs)/ In vivo (rat model)	Bone regeneration	PCR, histological and IHC analyses	[206]
miRNA (miR-122)	Commercial transfection reagents (not specified)	In vitro (rMSCs)/ In vivo (rat model)	Bone regeneration	PCR, WB, histological and IHC analyses	[180]
miRNA (antmiR-138)	Lipofectamine 2000^®^	In vitro (BMMSCs)/ In vivo (mice model)	Bone regeneration	PCR, WB, histological and IHC analyses	[117]
miRNA (antmiR-138)	Lipofectamine 2000^®^	In vitro (rat BMMSCs)	Bone regeneration	ALP assay, PCR, and CC analysis	[116]
siRNA (siNoggin)	Lipofectamine 2000^®^ and cationic stereosomes	In vitro (AMSCs)/ In vivo (mouse model)	Bone regeneration	ALP assay, PCR, and histological analysis	[118]
pDNA (RUNX2); siRNA (siOCT3, siOCT4 and siNANOG)	Poly (β-amino ester) polyplexes	In vitro (DPPSCs)	Bone regeneration	ALP assay, PCR, and ICC analysis	[26]
pDNA (BMP-2); siRNA (siNoggin)	C32-122 polyplexes and NA114 lipoplexes	In vitro (BMMSCs)	Bone regeneration	ALP assay, ELISA, PCR, Calcium Reagent kit, and CC analysis	[34]
siRNA (siNoggin); miRNA (miR-20a)	PEI polyplexes	In vitro (hMSCs)	Bone regeneration	CC analysis	[68]
siRNA (siNoggin); miRNA (miR-20a)	PEI polyplexes	In vitro (hMSCs)	Bone regeneration	ALP assay, PCR, Calcium Reagent kit, and CC analysis	[191]
pDNA (BMP-2 and TGF-β3)	nHA and PEI	In vitro (MSCs)	Osteochondral regeneration	ELISA, biochemical, histological and ICC analyses	[19]
pDNA (TGF-β1)	Gelatin microspheres	In vitro (hMSCs)	Chondrogenic differentiation	Biochemical, histological and ICC analyses	[1]
pDNA (TGF-β1)	Pullulan spermine polyplexes	In vitro (rMSCs)	Chondrogenic differentiation	CC analysis	[135]
pDNA (SOX9)	PEI-modified PLGA polyplexes	In vitro (hMSCs)	Chondrogenic differentiation	PCR, biochemical analysis, and immunoblotting	[27]
pDNA (Endostatin)	GP2^®^ lipoplexes	In vitro (cBMMSCs)	Chondrogenic differentiation	ELISA, and CC analysis	[188]
mRNA (SOX9 and MYOD)	3DFectIN^®^ lipoplexes	In vitro (hMSCs)	Chondrogenic and myogenic differentiation	PCR, and ICC analysis	[194]
pDNA (BMP-2 and TFG-β3)	Lipofectamine 2000^®^ lipoplexes	In vitro (hiPSCs)/ In vivo (rat model)	Osteochondral regeneration	PCR, histological and immunochemical analysis	[111]
pDNA (EGFP-C1)	PAA-BA and PEI polyplexes; Lipofectamine 2000® lipoplexes	In vitro (BMMSCs)	Multipotent differentiation	PCR, and ICC analysis	[120]
pDNA (BMP-2 and TGF-β3)	PEI polyplexes, HA nanoparticles, RALA peptide	In vitro (hMSCs)	Osteochondral differentiation	ELISA, PCR, biochemical, CC and ICC analyses	[179]
pDNA (BMP-2 and TGF-β1)	nHA (mineral-coated) and Lipofectamine 2000^®^ lipoplexes	In vitro (hMSCs)	Osteogenic differentiation	Biochemical and CC analyses	[114]
pDNA (BMP-2 and TGF-β3)	CaP nanoparticles or CaP/PEI polyplexes	In vitro (hMSCs)	Osteogenic differentiation	PCR, and CC analysis	[153]
pDNA (BMP-2 and TGF-β1)	Scaffold HA	In vivo (rabbit model)	Osteogenic differentiation	Histological analysis	[186]
miRNA (miR100-5p and miR143-3P)	PEI polyplexes	In vitro (MSCs)	Osteogenic differentiation	PCR, and CC analysis	[67]
miRNA (miR-133a)	nHA	In vitro (hMSCs)	Osteogenic differentiation	PCR, Calcium Liquicolor kit, and ICC analysis	[183]
miRNA (antmiR-138)	Chitosan/tripolyphosphate/HA nanoparticles	In vitro (MSCs)	Osteogenic differentiation	PCR, WB, and CC analysis	[184]
miRNA (miR-218)	*b*PEI polyplexes	In vitro (hMSCs)	Osteogenic differentiation	ALP assay, PCR, WB, and CC analysis	[182]

Abbreviations. *pDNA* plasmid DNA, *BMP-2, -6, -7, -9* bone morphogenic protein-2, -6, -7, -9, *PEI* polyethyleneimine, *BMMSCs* bone marrow derived mesenchymal stem cells, *FGF-2* fibroblast growth factor, *CC* cytochemical, *DPMSCs* dental pulp mesenchymal stem cells, *nHA* hydroxyapatite nanoparticles, *ELISA* enzyme-linked immunosorbent assay, *PCR* polymerase chain reaction, *VEGF* vascular endothelial growth factor, *rMSCs* rat mesenchymal stem cells, *GET* GAG-binding enhanced transduction, *TDMSCs* tonsil derived mesenchymal stem cells, *gMSCs* goat mesenchymal stem cells, *TGF-β1 and -β3* transforming growth factor-β1 and -β3, *ALP* alkaline phosphatase, *rAMSCs* rat adipose derived mesenchymal stem cells, *OSX* osterix, *GFP* green fluorescent protein, *bPEI* branched polyethyleneimine, *cDNA* circular DNA, *miRNA* micro-RNA, *siRNA* small interfering RNA, *RUNX2* core binding factor alpha 2, *OCT3 and 4* octamer-binding transcription factor 3 and 4, *DPPSCs* dental pulp pluripotent stem cells, *hMSCs* human mesenchymal stem cells, *SOX9* sex-determining region Y-type high mobility group box 9, *cBMMSCs* caprine bone marrow derived mesenchymal stem cells, *hiPSCs* human induced pluripotent stem cells, *MYOD* myogenic differentiation protein, *EGFP* enhanced green fluorescent protein, *CaP* calcium phosphate, *HA* hyaluronic acid, *PAA-BA* poly (amidoamine) with pendant aminobutyl group

MSCs play a crucial role in the bone-healing process as they serve as precursors to osteoblasts and chondrocytes, and they likely also contribute to modulating the healing response. Thus, the osteogenesis of MSCs is regulated by various proteins, hormones, and GFs. Most of the osteo-reparative approaches focus on the overexpression of NAs encoding for protein GFs, such as BMP-2 alone [5, 20–23, 115, 175, 177, 190], or in combination with other related family members, such as BMP-6 [65], BMP-7 [65], or BMP-9 to promote MSCs osteogenesis [218]. Although BMP-2 has a significant osteogenic potential, its activity can be hindered by the presence of extracellular inhibitors and antagonists, such as Noggin, which exhibits a strong affinity for BMP-2 and prevents it from binding to its receptor. In order to enhance the effects of BMP-2, the use of siRNA against these regulators has therefore emerged as a promising approach. This technique aims to suppress the expression of these inhibitors, thus allowing for a more potent and effective action of BMP-2. Hence, an effective osteogenic differentiation of MSCs can be attained using siRNA against Noggin [34, 68, 118, 191]. Moreover, a variety of miRNAs, including anti miR-138, miR-20a, miR-218, and miR-133a [67, 68, 116, 117, 180, 183, 184, 191, 206], have been identified as potential targets to upregulate cell signaling pathways related to osteogenesis.

Specific culture conditions are also required to selectively induce osteo- or chondrogenic differentiation of MSCs after transfection with suitable therapeutic genes. Of note, most protocols in this regard require a minimum culture time of 14 days, and the use of media enriched with ascorbic acid, β-glycerophosphate, and dexamethasone [194].

Commonly assessed osteogenic markers comprise RUNX2, osterix (OSX), ALP, COL-I, osteocalcin (OCN), and a range of members from the BMP superfamily. Because ALP, BMPs, COL-I, RUNX2, and OSX are essential for osteoblast differentiation, they are considered early markers of osteogenesis. Instead, OCN and osteopontin (OPN) are deemed late cell differentiation markers as they are involved in mineralization and ECM synthesis. Since the expression levels of such biomarkers increase in the sequential steps of bone formation and regeneration, their monitoring over time employing analytical techniques such as qPCR [18, 20, 26, 67, 116, 117, 152, 177, 180, 183, 190, 202, 206, 218], ELISA [19, 21–23, 115, 178, 202, 203] or WB [20, 117, 180, 184, 191] is a valuable way to assess the effectiveness of a given osteogenic strategy.

The detection of biochemical markers associated with the differentiation process can be also performed through spectrophotometric measurements. Osteogenic markers can be assessed by measuring ALP expression and calcium deposition by the cells. ALP is the most recognized protein marker of early osteogenic differentiation due to its pivotal role in bone matrix formation and calcification [5, 18, 23, 65, 116–118]. Several kits are available to detect and quantify this protein. They rely on the use of substrates that are converted by the secreted ALP into colored, fluorescent, or luminescent products. Besides, to determine the calcium concentration within the physiological range, there are colorimetric kits (λ (Abs) = 575 nm) that detect alkaline phosphatase substrate (pNPP-Na hexahydrate) in cell samples.

In addition to the aforementioned techniques, IHC and ICC are mostly used to detect osteogenic differentiation, as they offer the possibility of investigating the distribution of an antigen over the different cell types thus allowing to gather insights on the tissue architecture. Specific histological stains for bone encompass Alizarin red [5, 18, 65, 68, 69, 116, 118, 174, 175, 190, 191, 202, 206, 218] and Sirius red [117, 180, 194], which stain in red–orange the calcium deposits and the collagen fibers, respectively.

The process of cartilage development begins with the condensation of MSCs, followed by their differentiation into chondrocytes, which secrete the cartilage matrix components such as proteoglycans and COL-II. Of note, it is important to maintain an appropriate balance of these markers during cartilage formation to prevent chondrocytes hypertrophy and matrix mineralization, as an imbalance can compromise the integrity of the cartilage tissue [221].

For this reason, common strategies to promote the chondrogenic differentiation of MSCs rely on the use of NAs encoding for members of the TGF-β superfamily, such as TGF-β1 or TGF-β3, which enhanced the expression of proteoglycans and COL-II. However, the main limitation of this factor is that often results in the expression of hypertrophic and osteogenic markers [222]. Additionally, the cartilage-specific transcription factor SOX9 holds potential in achieving better outcomes for cartilage regeneration, promoting chondrocytes proliferation and the cartilage matrix synthesis while minimizing hypertrophy [131].

Besides, the chondrogenic differentiation of MSCs is quite often conducted in 3D cultures (aggregate or matrix) for a minimum of 21 days, using media containing ascorbic acid, dexamethasone, insulin-transferrin-selenious acid mix, pyruvate, bovine serum albumin (BSA) and TGF-β [111, 194].

**Fig. 6:**
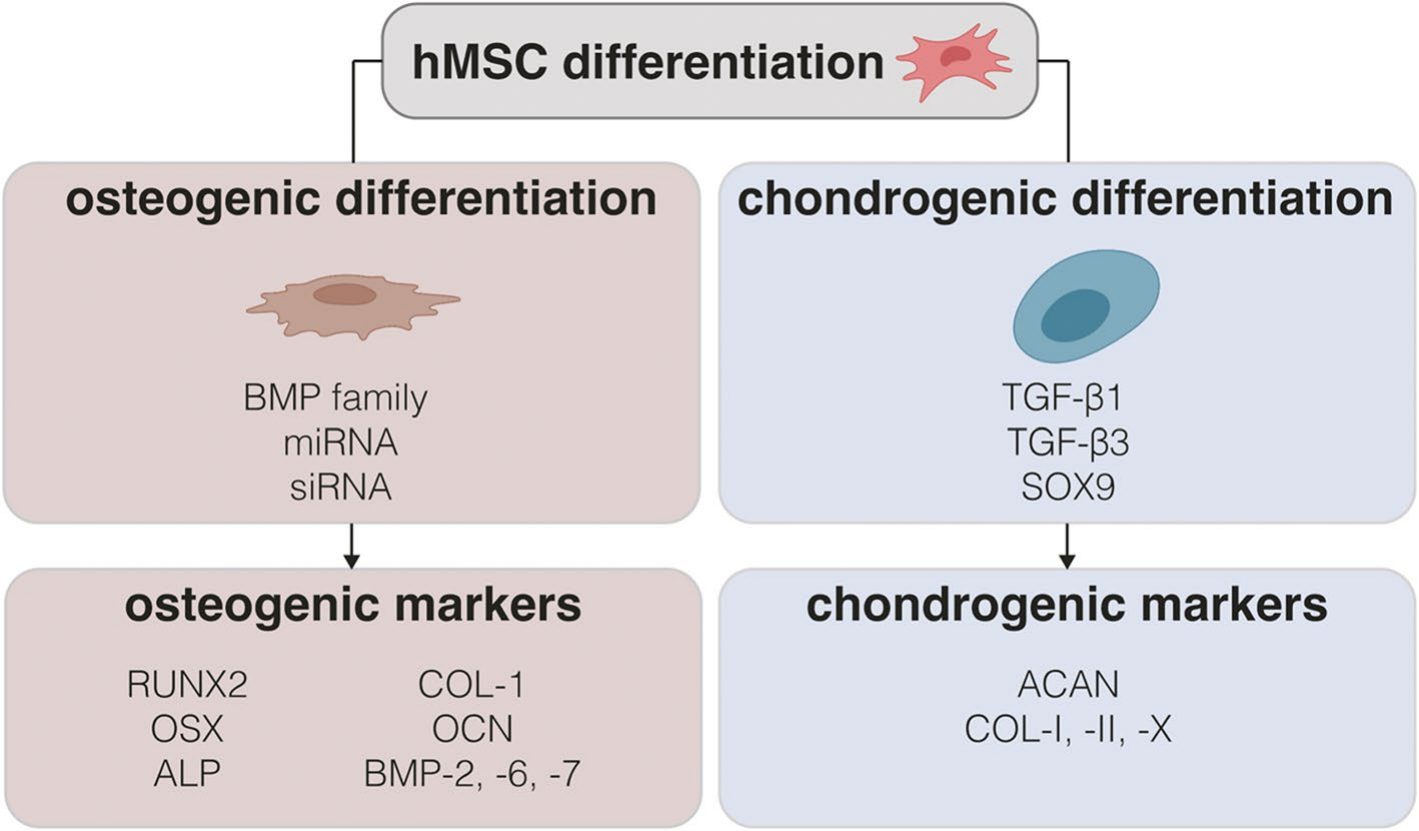
Most used therapeutic genes and markers to induce and evaluate osteochondral differentiation. Abbreviations: BMP-2, -6, -7, -9: bone morphogenic protein 2, 6, 7, 9; siRNA: small interfering RNA; miRNA: micro-RNA; TGF-β1 and -β3: transforming growth factor-β1 and -β3; SOX9: sex-determining region Y-type high mobility group box 9; RUNX2: core binding factor alpha 2; OSX: osterix; ALP: alkaline phosphatase; COL-I, -II, -X: type-I, -II, and -X: collagen; OCN: osteocalcin; ACAN: aggrecan

Gene markers of chondrogenesis include aggrecan (ACAN), COL-I, COL-II, COL-X, and the early chondrogenic factor SOX9. However, their expression patterns are specific to the cartilage type. For instance, the upregulation of ACAN and COL-II genes often occurs during articular cartilage repair, whereas the expression of COL-I (fibrocartilage marker) and COL-X (hypertrophic marker) might decrease (Fig. 6). Similar to the osteogenic processes, these markers can be evaluated by qPCR [27, 111, 120, 153, 194], ELISA [179, 188] or WB [27] techniques.

Common biochemical analysis of chondrogenesis involves the use of dyes such as dimethyl methylene blue (DMMB, λ Abs = 525 nm), which binds to sulfated GAGs enabling their prompt detection [1, 27, 114].

Histological analysis of cartilage phenotype usually relies on the staining of sulfated glycosaminoglycans (GAG) in the ECM with Alcian [27, 111, 135, 153, 179, 194] and Toluidine blue [111] (blue staining) or Safranin-O [1, 27, 111, 114, 135] (red staining). Nevertheless, the most reliable way to assess the osteo- or chondrogenic commitment of MSCs post-transfection is to combine at least two of the abovementioned methods.

## Conclusions and future perspectives

The use of MSCs as therapeutic vehicles holds great promise for the treatment of osteochondral diseases. MSCs can be easily isolated from different sources and manipulated ex vivo to direct them toward osteo- or chondrogenic lineages. However, the current approaches, mostly relying on the use of soluble factors to drive MSCs differentiation, are somehow frustrating and ineffective. In this scenario, the control of MSCs phenotype using non-viral gene delivery strategies has garnered increased interest in recent years. The transient expression achieved through these systems aligns more closely with natural wound healing processes, making them particularly appealing [223].

While continuous effort in gene delivery research has led to the development of safe and fair efficient vectors, no standard procedure does exist for the ex vivo engineering of hMSCs, thus limiting the translation of MSCs-based therapies into the clinics. In light of this, this review aims at highlighting the main progress undertaken in non-viral gene delivery research for MSCs’ transfection and suggest ways to improve their efficacy in the target cells. Since gene transfer outcomes strongly depend on the cell response, we pointed out the use of exogenous environmental cues (either mechanical stimuli or substrate features), combined with the delivery of non-viral vectors, as a way to improve gene transfer efficiency and drive MSCs differentiation.

Besides, since there is no real advancement without robust models, we also provided readers with a practical guide and some genuine results for conducting screening studies on MSCs. This allows for the evaluation of MSCs’ commitment to osteo and chondrogenic phenotypes in vitro. As part of this approach, we provide an exemplification of how to optimize transfection conditions with the gold standard cationic polymer PEI in primary cultures of MSCs.

When focusing on MSC differentiation assays, it is crucial to select the most appropriate therapeutic gene based on the desired application. Overexpression of the GF BMP-2, along with the utilization of siRNA to prompt osteogenesis-related cell signaling pathways, emerged as a promising strategy. In addition, the overexpression of the transcription factor SOX9 appears to be a suitable approach for enhancing MSCs chondrogenesis, leading to the development of through a hyaline-like cartilage phenotype while reducing hypertrophy.

Overall, through this comprehensive review, our sincere hope is to establish a solid foundation for advancing research in MSCs therapy, paving the way for further breakthroughs in this field.

## Supplementary Information

**Additional file 1.**

## Data Availability

Please contact author for data requests.

## Abbreviations

7-AAD: 7-Aminoactinomycin D
ACAN: Aggrecan
ALP: Alkaline phosphatase
baf-A1: Bafilomycin-A1
BMMSCs: Bone marrow derived mesenchymal stem cells
BMP-2, -6, -7, -9: Bone morphogenic protein-2, -6, -7, -9
bPEI: Branched polyethyleneimine
CaP: Calcium phosphate
cBMMSCs: Caprine bone marrow derived mesenchymal stem cells
CC: Cytochemical
cDNA: Circular DNA
CLs: Cationic lipids
CME: Clathrin-mediated endocytosis
CPs: Cationic polymers
COL-I: Type-I collagen
COL-II: Type-II collagen
COL-X: Type-X collagen
CSM: Confocal microscopy
CvME: Caveolae-mediated endocytosis
Cy3: Cyanine 3
Cy5: Cyanine 5
DAPI: 4', 6-Diamidino-2-phenylindol
DEX: Dexamethasone
DMMB: Dimethyl methylene blue
DOPE: 1,2-Dioleoyl-sn-glycerol-3-phosphoethanolamine
DOSPA: Cationic lipid 2,3-dioleoyloxy-N-[2(sperminecarboxiamido)ethyl[-N,N-dimethyl-1-propaniminium trifluoroacetate
DPMSCs: Dental pulp mesenchymal stem cells
DPPSCs: Dental pulp pluripotent stem cells
ECM: Extracellular matrix
EGFP: Enhanced green fluorescent protein
ELISA: Enzyme-linked immunosorbent assay
FCM: Flow cytometry
FITC: Fluorescein isothiocyanate
FGF-2: Fibroblast growth factor
GAG: Glycosaminoglycans
GET: GAG-binding enhanced transduction
GFs: Growth factors
GFP: Green fluorescent protein
gMSCs: Goat mesenchymal stem cells
HA: Hyaluronic acid
hAMSCs: Human adipose mesenchymal stem cells
hBMMSCs: Human bone marrow derived mesenchymal stem cells
hiPSCs: Human induced pluripotent stem cells
hMSCs: Human mesenchymal stem cells
hUCMSCs: Human umbilical cord mesenchymal stem cells
ICC: Immunocytochemistry
IGF-1: Insulin-like growth factor-1
IHC: Immunohistochemistry
luc: Luciferase
MCM: Mineral-coated microparticles
Me-β-CD: Methyl-β-cyclodextrin
miRNA: Micro-RNA
MYOD: Myogenic differentiation protein
NAs: Nucleic acids
nHA: Hydroxyapatite nanoparticles
NPs: Nanoparticles
OCN: Osteocalcin
OCT3 and 4: Octamer-binding transcription factor 3 and 4
OPN: Osteopontin
OSX: Osterix
PAA-BA: Poly (amidoamine) with pendant aminobutyl group
PAMAM: Polyamidoamines
PCR: Polymerase chain reaction
pDNA: Plasmid DNA
PEI: Polyethyleneimine
PI: Propidium iodide
PLGA: Poly (DL-lactic-co-glycolic acid)
PLLs: Poly-L-lysines
rAMSCs: Rat adipose derived mesenchymal stem cells
RFP: Red fluorescence protein
RITC: Rhodamine isothiocyanate
rMSCs: Rat mesenchymal stem cells
RUNX2: Runt-related transcription factor 2
SAR: Structure–function relationship
siRNA: Small interfering RNA
SOX9: Sex-determining region Y-type high mobility group box 9
TDMSCs: Tonsil derived mesenchymal stem cells
TGF-β1 and -β3: Transforming growth factor-β1 and -β3
UV: Ultraviolet
VEGF: Vascular endothelial growth factor
WB: Western blot
YFP: Yellow fluorescence protein
