# Supplementary material for: Non-viral gene delivery to human mesenchymal stem cells: a practical guide towards cell engineering
Source: J Biol Eng. 2023 Jul 25;17:49. doi: 10.1186/s13036-023-00363-7 (PMC10369726; doi:10.1186/s13036-023-00363-7)

**Supplementary Material**

**Non-viral gene delivery to human mesenchymal stem cells: a practical guide towards cell engineering**

*Natalia Carballo-Pedrares^1†^, Federica Ponti^2,3†,^ Junquera Lopez-Seijas^1^, Diego Miranda-Balbuena^1^, Nina Bono^2^, Gabriele Candiani^2^*, Ana Rey-Rico**

*^1^* Gene & Cell therapy research group (G-CEL). Centro Interdisciplinar de Química e Bioloxía - CICA, Universidade da Coruña, As Carballeiras, s/n. Campus de Elviña, 15071 A Coruña, Spain.

*^2^* genT_LΛB, Department of Chemistry, Materials and Chemical Engineering “G. Natta”, Politecnico di Milano, 20131 Milan, Italy.

*^3^* Laboratory for Biomaterials and Bioengineering, CRC Tier I, Department of Min-Met-Mat Engineering and CHU de Québec Research Center, Division of Regenerative Medicine, Laval University Québec, QC, Canada

**†** These authors equally contributed to this work

**Corresponding Authors**

Gabriele Candiani*^2^**

genT_LΛB, Department of Chemistry, Materials and Chemical Engineering “G. Natta”, Politecnico di Milano, 20131 Milan, Italy

Phone: +39 02 2399 3181

e-mail: [gabriele.candiani@polimi.it](mailto:gabriele.candiani@polimi.it)

Ana Rey-Rico^1^*

*^1^* Centro de Investigacións Científicas Avanzadas (CICA), Universidade da Coruña, 15071 A Coruña, Spain.

Phone: +34 881 01 5543

e-mail: [ana.rey.rico@udc.es](mailto:ana.rey.rico@udc.es)

**Table of contents**

[**1. Materials** 3](#_Toc119514202)

[**2. Methods** 3](#_Toc119514203)

[**2.1. Plasmid propagation and purification** 3](#_Toc119514204)

[**2.2. Preparation of transfectant solution** 3](#_Toc119514206)

[**2.3. *In vitro* transfection experiments** 3](#_Toc119514207)

[**2.3.1. Cell culture** 3](#_Toc119514208)

[**2.3.2. *In vitro* cell transfection assays** 4](#_Toc119514209)

[**2.3.3. Evaluation of cytotoxicity** 4](#_Toc119514210)

[**2.3.4. Evaluation of transfection efficiency** 4](#_Toc119514211)

[**2.3.5. Statistical analysis** 5](#_Toc119514212)

[**3. References** 5](#_Toc119514213)

[**Figure S1**. 6](#_Toc119514214)

# **Materials**

pGL4.14 (plasmid DNA - pDNA - encoding the luciferase reporter gene *luc2* (*Photinus pyralis*), 5,809 bp) and Luciferase Assay System was purchased from Promega (Madison, WI, USA), while pDNA purification kit was from Roche (Mannheim, Germany). 25 kDa branched polyethyleneimine (*b*PEI, cat. nr. 408727) was from Merck Life Science (Madrid, Spain). Any other reagents were purchased from Gibco-ThermoFisher Scientific (Madrid, Spain) unless otherwise stated.

# **Methods**

# **Plasmid preparation**

The top 10 competent cells were transformed with the plasmid pGL4.14 encoding the luciferase reporter gene *luc2* (hereafter referred to as p*luc*) and amplified at 37 °C under shaking. The pDNA was isolated and purified using a Maxiprep Roche kit according to the manufacturer’s instructions. The plasmid was next diluted at a concentration of 250 ng/µL in DNase-free water, then the concentration and purity were assessed by measuring the OD_260_/OD_280_ using a spectrophotometer (mySPEC, VWR, Barcelona, Spain). The pDNA solution was stored at -20 ºC until use.

# **Preparation of transfectant solution**

An aqueous solution of 25 kDa *b*PEI was prepared in deionized water (dH_2_O) at a concentration of 1 mg/mL, and the pH was adjusted to 7.0. Afterward, the solution was diluted in Opti-MEM^TM^ at a final concentration of 0.86 mg/mL, corresponding to an amine concentration ([N]) of 20 mM, considering that there is one nitrogen per repeat PEI unit (-NHCH_2_CH_2_-, M_W_ = 43 Da). The *b*PEI stock solution was stored at 4 ºC until use.

# ***In vitro* transfection experiments**

# **Cell culture**

Bone marrow aspirates were obtained from the proximal femur of patients undergoing hip arthroplasty (n = 2) provided by the Biobanco of A Coruña from SERGAS. The study was approved by the Comité de Ética de Investigación da Coruña (accession number: 2021/425). All patients provided informed consent before inclusion in the study. Human mesenchymal stem cells (hMSCs) were isolated and expanded in culture using standard protocols [1] and maintained in Dulbecco’s Modified Eagle’s Medium (DMEM), supplemented with 10 % (v/v) FBS, 100 U/mL penicillin G, 100 µL/mL streptomycin (hereafter referred to as complete medium) at 37 °C in a humidified atmosphere under constant supply of 5 % (v/v) CO_2_ (hereafter referred to as standard culture conditions).

# ***In vitro* cell transfection assays**

For any transfection experiments, hMSCs were kept between passages 1 and 2. hMSCs were seeded onto 96 well-plates at different cell densities, namely 5 × 10^3^, 1.5 × 10^4^, 5 × 10^4^, and 1 × 10^5^ cells/cm^2^, and incubated for 24 hrs in standard culture conditions. Twenty-four hours post-seeding, pDNA at different doses, namely 0.1 µg/cm^2^, 0.5 µg/cm^2^, or 1 µg/cm^2^, was complexed with *b*PEI*.* Briefly*,* an appropriate volume of *b*PEI solution was mixed with different doses of plasmid in Opti-MEM^TM^ to invariably yield N/P 10, followed by a 30 min-incubation at room temperature (r.t.). N/P is defined as the amine moles (N, cationic moiety) of the polymer with respect to the phosphate moles (P, anionic moiety) of a given quantity of pDNA [2]. Cells were challenged with polyplexes, then cultured in complete medium in standard culture conditions for 24, 48, and 72 hrs.

# **Evaluation of cytotoxicity**

At given time points post polyplex delivery, namely 24, 48, and 72 hrs, the cytotoxicity was evaluated using the Alamar Blue^®^ assay, according to the manufacturer’s instructions. Briefly, the complete medium was discarded, and each well was filled with 100 µL/well of 1× resazurin dye solution in the fresh complete medium. Plates were incubated in the dark, in standard culture conditions for 2 hrs, then the fluorescence was read using a Synergy HTX Plate Reader (λ_ex_ = 485 nm; λ_em_ = 528 nm; Biotek, Winooski, VT, USA). The viability of untransfected cells (CTRL) was assigned to 100 %, and the viability of transfected samples was determined as follows (eq. 1):

Viability (%) = $\frac{F_{\mathrm{sample}}}{F_{\mathrm{CTRL}}} \times100$ (eq. 1)

Where F is the recorded fluorescence.

# **Evaluation of transfection efficiency**

The transgene expression was evaluated at given time points, namely 24, 48, and 72 hrs from polyplex delivery, by measuring the luciferase activity in cell lysates using the Luciferase Assay system, following the manufacturer’s instructions. Briefly, 20 µL of cell lysate was mixed with 50 µL of luciferase assay substrate. The luminescence signal (Relative Light Units, RLU) was normalized to the total protein content of each sample, as determined by the BCA assay. Transfection efficiency was expressed as RLU/mg of protein.

# **Statistical analysis**

Statistical analysis was performed using IBM SPSS Statistics version 23 and R Studio. Data were initially analyzed using D’Agostino & Pearson omnibus normality tests. Comparison between groups was performed with a three-way analysis of variance (ANOVA). Significance was retained when p < 0.05. Data are expressed as mean ± standard deviation (SD). Experiments were performed at least twice using cells isolated from two different patients. For each experiment, n = 3 technical replicates were performed.

# **References**

[1] Cucchiarini M, Ekici M, Schetting S, Kohn D, Madry H. Metabolic Activities and Chondrogenic Differentiation of Human Mesenchymal Stem Cells Following Recombinant Adeno-Associated Virus-Mediated Gene Transfer and Overexpression of Fibroblast Growth Factor 2. *Tissue EngineeringPart A* 17(15-16), 1921-1933 (2011).

[2] Bono N, Ponti F, Mantovani D, Candiani G. Non-Viral in Vitro Gene Delivery: It Is Now Time to Set the Bar! *Pharmaceutics* 12(2), 183 (2020).

**Figure S1**. Cell viability is the percent cell viability (%), as compared to untransfected cells (CTRL), following transfection with *b*PEI/p*luc* complexes prepared at N/P 10 on hMSCs isolated from two patients (i.e., patient #1 and patient #2, right and left panels, respectively) as a function of the pDNA dose (0.1, 0.5, and 1 µg/cm^2^) and the cell density (5 × 10^3^, 1.5 × 10^4^, 5 × 10^4^, and 1 × 10^5^ cells/cm^2^). Cell viability was evaluated 24 (black), 48 (white), and 72 hrs (grey) post-transfection. Results are expressed as mean ± SD (n = 3).


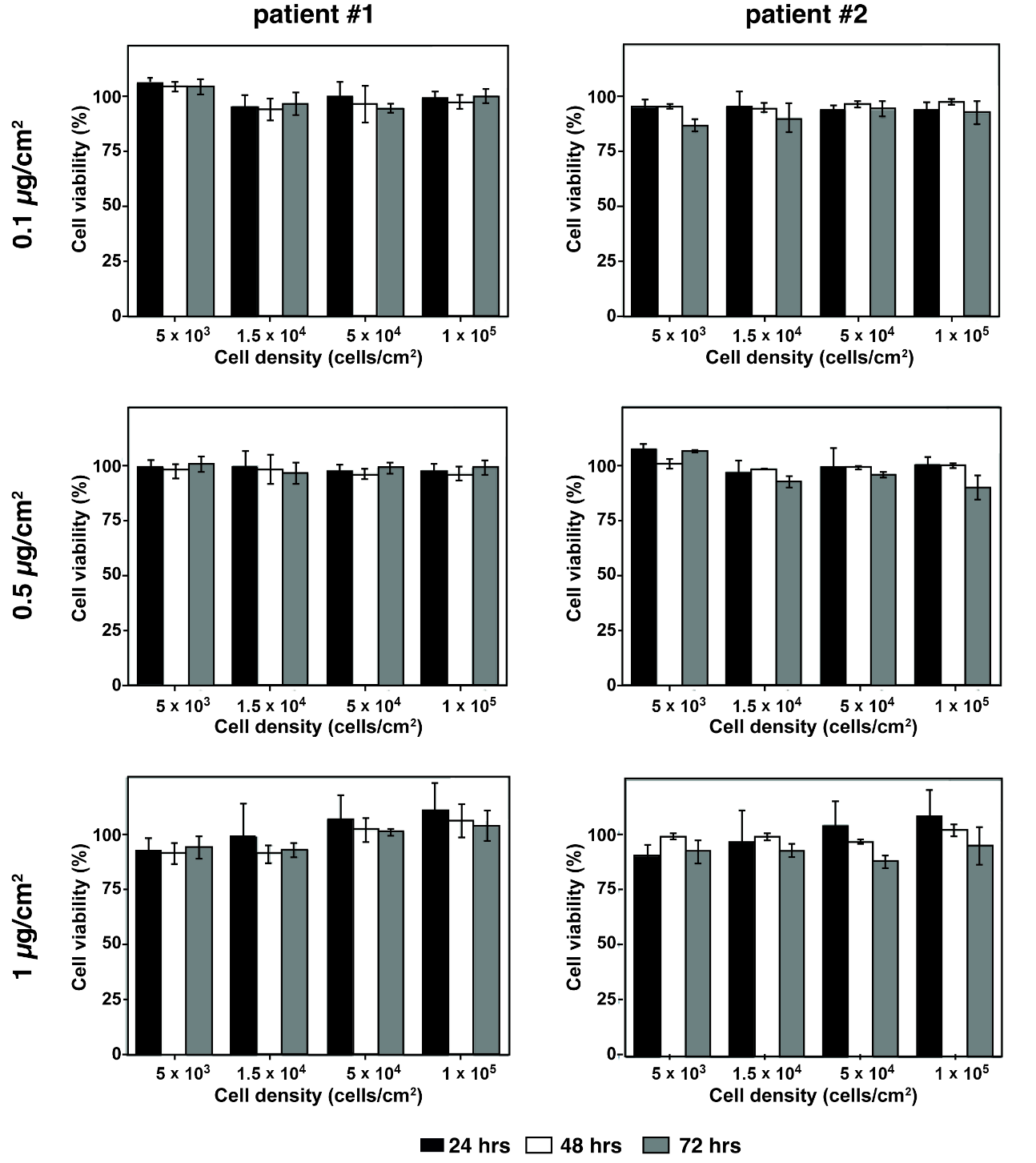

Supplement: Supplementary file 1 — Additional file 1. [file 13036_2023_363_MOESM1_ESM.docx]
